# Rapid forest carbon assessments of oceanic islands: a case study of the Hawaiian archipelago

**DOI:** 10.1186/s13021-015-0043-4

**Published:** 2016-01-08

**Authors:** Gregory P. Asner, Sinan Sousan, David E. Knapp, Paul C. Selmants, Roberta E. Martin, R. Flint Hughes, Christian P. Giardina

**Affiliations:** 1 Department of Global Ecology, Carnegie Institution for Science, 260 Panama St, Stanford, CA 94305 USA; 2 Department of Natural Resources and Environmental Management, University of Hawaii at Manoa, 1910 East–West Rd., Honolulu, HI 96822 USA; 3 USDA Forest Service, Pacific Southwest Research Station, Institute of Pacific Islands Forestry, 60 Nowelo Street, Hilo, HI 96720 USA

**Keywords:** Carbon stocks, Carnegie Airborne Observatory, Forest inventory, Invasive species, LiDAR, Random Forest Machine Learning

## Abstract

**Background:**

Spatially explicit forest carbon (C) monitoring aids conservation and climate change mitigation efforts, yet few approaches have been developed specifically for the highly heterogeneous landscapes of oceanic island chains that continue to undergo rapid and extensive forest C change. We developed an approach for rapid mapping of aboveground C density (ACD; units = Mg or metric tons C ha^−1^) on islands at a spatial resolution of 30 m (0.09 ha) using a combination of cost-effective airborne LiDAR data and full-coverage satellite data. We used the approach to map forest ACD across the main Hawaiian Islands, comparing C stocks within and among islands, in protected and unprotected areas, and among forests dominated by native and invasive species.

**Results:**

Total forest aboveground C stock of the Hawaiian Islands was 36 Tg, and ACD distributions were extremely heterogeneous both within and across islands. Remotely sensed ACD was validated against U.S. Forest Service FIA plot inventory data (R^2^ = 0.67; RMSE = 30.4 Mg C ha^−1^). Geospatial analyses indicated the critical importance of forest type and canopy cover as predictors of mapped ACD patterns. Protection status was a strong determinant of forest C stock and density, but we found complex environmentally mediated responses of forest ACD to alien plant invasion.

**Conclusions:**

A combination of one-time airborne LiDAR data acquisition and satellite monitoring provides effective forest C mapping in the highly heterogeneous landscapes of the Hawaiian Islands. Our statistical approach yielded key insights into the drivers of ACD variation, and also makes possible future assessments of C storage change, derived on a repeat basis from free satellite data, without the need for additional LiDAR data. Changes in C stocks and densities of oceanic islands can thus be continually assessed in the face of rapid environmental changes such as biological invasions, drought, fire and land use. Such forest monitoring information can be used to promote sustainable forest use and conservation on islands in the future.

**Electronic supplementary material:**

The online version of this article (doi:10.1186/s13021-015-0043-4) contains supplementary material, which is available to authorized users.

## Background

Aboveground carbon (C) stock assessments have become a mainstay of forest management [1]. In the past decade, the importance of such assessments has also grown in the climate change mitigation arena [2]. In step with these efforts, there has been increasing focus on developing quantitative methods to monitor forest C stocks over time, as a means to support policies that reduce emissions from deforestation and forest degradation, and increase C storage in existing forests (REDD+) [3]. C storage has also become an important metric for assessing forest habitat and condition in the broader conservation arena [4, 5].

Based on the increasing value in understanding the geography of forest C stocks, both field-based and remote sensing-assisted C assessments have been undertaken over larger and larger geographic areas [6, 7]. Far less attention, however, has been given to oceanic islands, likely due to their relatively small land area. Oceanic islands provide model socio-ecological systems with which to examine spatial patterns in forest C stocks, because islands are often comprised of highly heterogeneous ecosystems, where many of the drivers of C storage (e.g., vegetation types, climate, fire, and land use) vary strongly over short distances [8, 9]. While C stocks on oceanic islands may be small in a global context, they provide unique opportunities to test fundamental concepts on the landscape ecology, sociology, economics and management of forest C sequestration. Further, forests on oceanic islands are quite important to the provisioning of ecosystem goods and services, including fresh water supply, prevention and mitigation of soil erosion that can deplete upland soil resources and pollute aquatic ecosystems including coral reefs [10], and both timber and non-timber forest products. Island forests also play a strong cultural role as a locus of subsistence and recreational activities [11, 12]. However, relative to continental ecosystems, forests on oceanic islands continue to undergo a much greater proportional extent and rate of change in cover and composition, which threatens the sustainability of forest-based good and services including C stocks [13, 14]. Not only have islands been heavily deforested in some regions of the world, they have also undergone enormous change via introduced disturbance regimes, such as fire, and alien invasive species [15, 16]. The effects of these and other changes on forest C stocks remain poorly understood, despite numerous local- to landscape-scale assessments [17]. Without continuous and spatially extensive forest monitoring, patterns of change and/or opportunities for recovery of island forests will remain a challenge to incorporate into conservation, management and resource policy initiatives.

Like most oceanic islands, aboveground forest C stocks within and across the Hawaiian Islands are poorly known, owing to extreme environmental heterogeneity combined with local inaccessibility and complex terrain. This has greatly limited efforts to develop and maintain operational, repeat forest inventory on the ground. Global remote sensing-based carbon mapping approaches generally yield lower spatial resolutions and C stock sensitivities [18–21], which are difficult to apply in regions of high ecological heterogeneity like islands. While high-resolution remote sensing methods, such as airborne Light Detection and Ranging (LiDAR) [22], are suitable for such settings [23], mapping remote or difficult-to-access areas with aircraft can be expensive. In particular, cloud cover is often persistent over higher-elevation forests of key interest in forest C and watershed assessments. As a result, airborne campaigns can be prolonged and accumulate costs. An added challenge is that island forest assessments are needed on a repeat basis in response to the inherent vulnerability of many island landscapes to rapid change driven by land use, fire, storms (e.g., hurricanes), biological invasions and sea level rise. The issue of rapid change calls for the development of a low-cost, repeatable forest monitoring method for island forests. Such rapid, high-resolution assessment capabilities must be sensitive to the drivers of forest C change, not only as a metric for climate change mitigation, but also as a measure of forest health and provisioning services.

While mapping of forest C stocks has been challenged by uncertainty and cost [7], recent progress at subnational to national levels indicates that significant methodological hurdles can be overcome at larger scales, especially through the fusion of ground, aircraft and satellite based measurements [21, 24]. These approaches can simultaneously increase map resolution in ways that benefit forest managers, while reducing uncertainty to levels acceptable to policy makers. Despite these advances, important methodological questions remain regarding how to provide high resolution, low uncertainty monitoring at low cost in heterogeneous landscapes. A further need is the simultaneous assessment of the drivers of spatial variation in C storage.

**Fig. 1:**
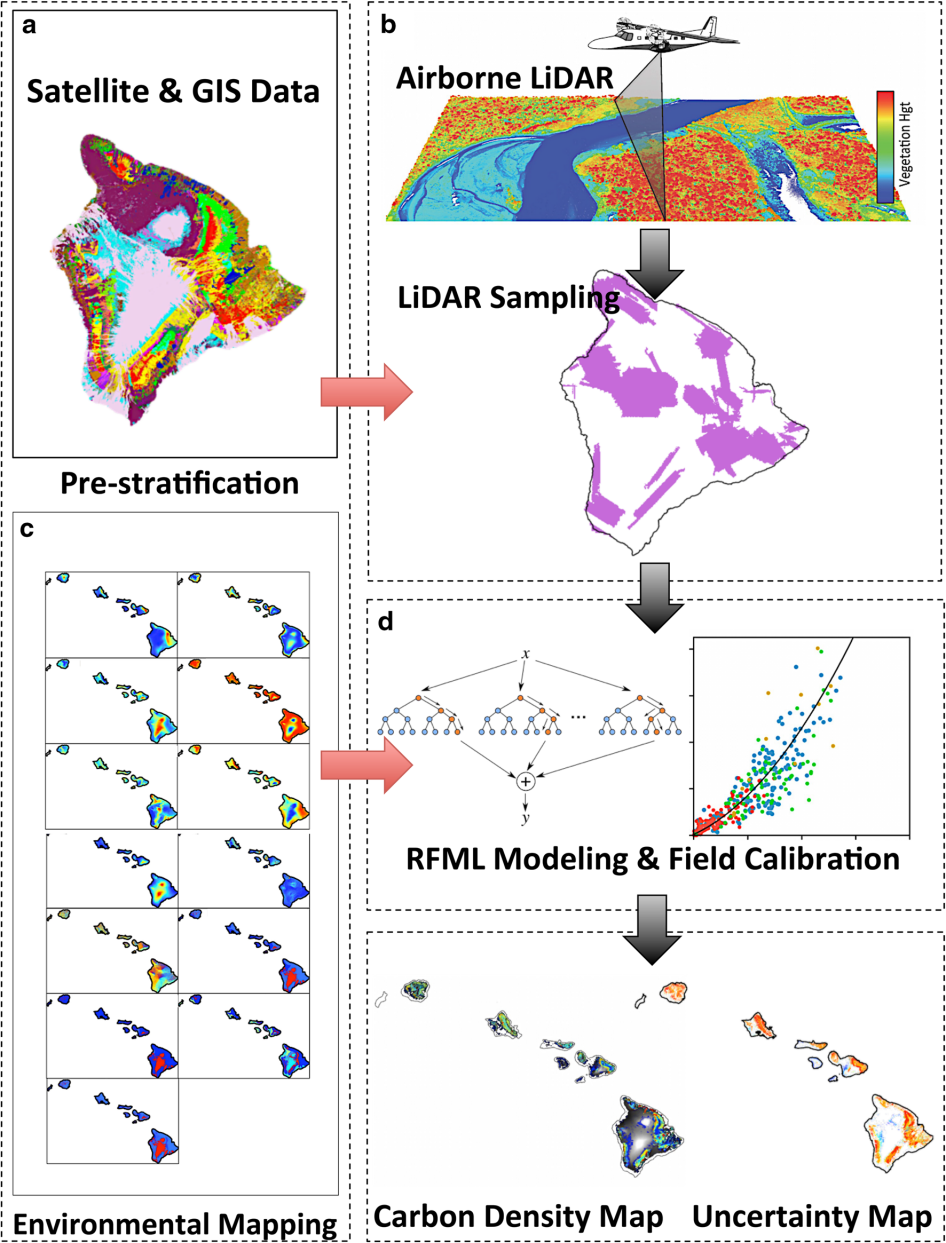
Overview of the methodology used to map vegetation carbon stocks throughout Hawaii: **a**, **b** the Hawaii State GAP vegetation map [34] provided a geospatial guide for sampling Hawaii Island with airborne Light Detection and Ranging (LiDAR). The LiDAR data were converted to maps of top-of-canopy height (TCH). **c** A diverse array of satellite-based environmental maps were compiled to provide continuous geographic information on vegetation cover, topographic variables, and climate. **d** The satellite and LiDAR data were processed through a geostatistical model based on the Random Forest Machine Learning (RFLM) approach [54] to develop multi-island, statewide maps of TCH at 30 m spatial resolution. The statewide TCH map was converted to estimates of aboveground carbon density (ACD) using a universal plot-aggregate approach [26]. The modeling process included an estimate of uncertainty on each 30 m grid cell for the entire State of Hawaii

We developed an approach for monitoring forest aboveground carbon density (ACD; units = Mg or metric tons C ha^−1^) across island archipelagos at a spatial resolution of 30 m (0.09 ha) using a combination of airborne LiDAR and freely available satellite data (Fig. 1). The approach involves initial use of high-resolution LiDAR sampling of a selected island within an archipelago to derive vegetation canopy height data. These data from the sampled island are then used to train a geospatial model that incorporates maps of multiple environmental factors, as well as forest canopy structural metrics derived from Landsat or comparable satellite imagery [25]. The resulting model is applied to all islands within the archipelago using as input the same portfolio of environmental and satellite-based canopy structural maps used on the model-training island, thereby yielding a multi-island map of canopy height at 30-m spatial resolution. Finally a regionally-tuned equation is applied to relate mapped canopy height to ACD [26], resulting in a carbon density map at 30-m resolution for the entire island chain. Critically, once the model is built for an archipelago, subsequent changes in ACD can be detected using only Landsat imagery, thereby greatly reducing longer-term monitoring costs [24].

For this study, we first sampled Hawaii Island, by far the largest island in the Hawaiian archipelago, with airborne LiDAR to assess forest top-of-canopy height (TCH) responses to natural environmental gradients and land use (Additional file 1: Figure S1). These LiDAR TCH data from Hawaii Island were used to calibrate a Random Forest Machine Learning (RFML) model, which was subsequently used to predict TCH at 30 m resolution on all islands from a portfolio of spatially explicit predictor maps (Additional file 1: Figures S2-S4). The resulting statewide model of forest TCH was then used to estimate forest ACD via a conversion equation developed for the Hawaiian Islands (Additional file 1: Figure S5). The resulting map was compared to US Forest Service Forest Inventory and Analysis (FIA—http://www.fia.fs.fed.us/) plot data for evaluation of mapped ACD precision. Finally, we used the new ACD map to assess aboveground forest C stocks within and among islands, in protected and unprotected areas, and among forests dominated by native and invasive plant species.

## Results and discussion

### Island carbon stocks and distributions

**Fig. 2:**
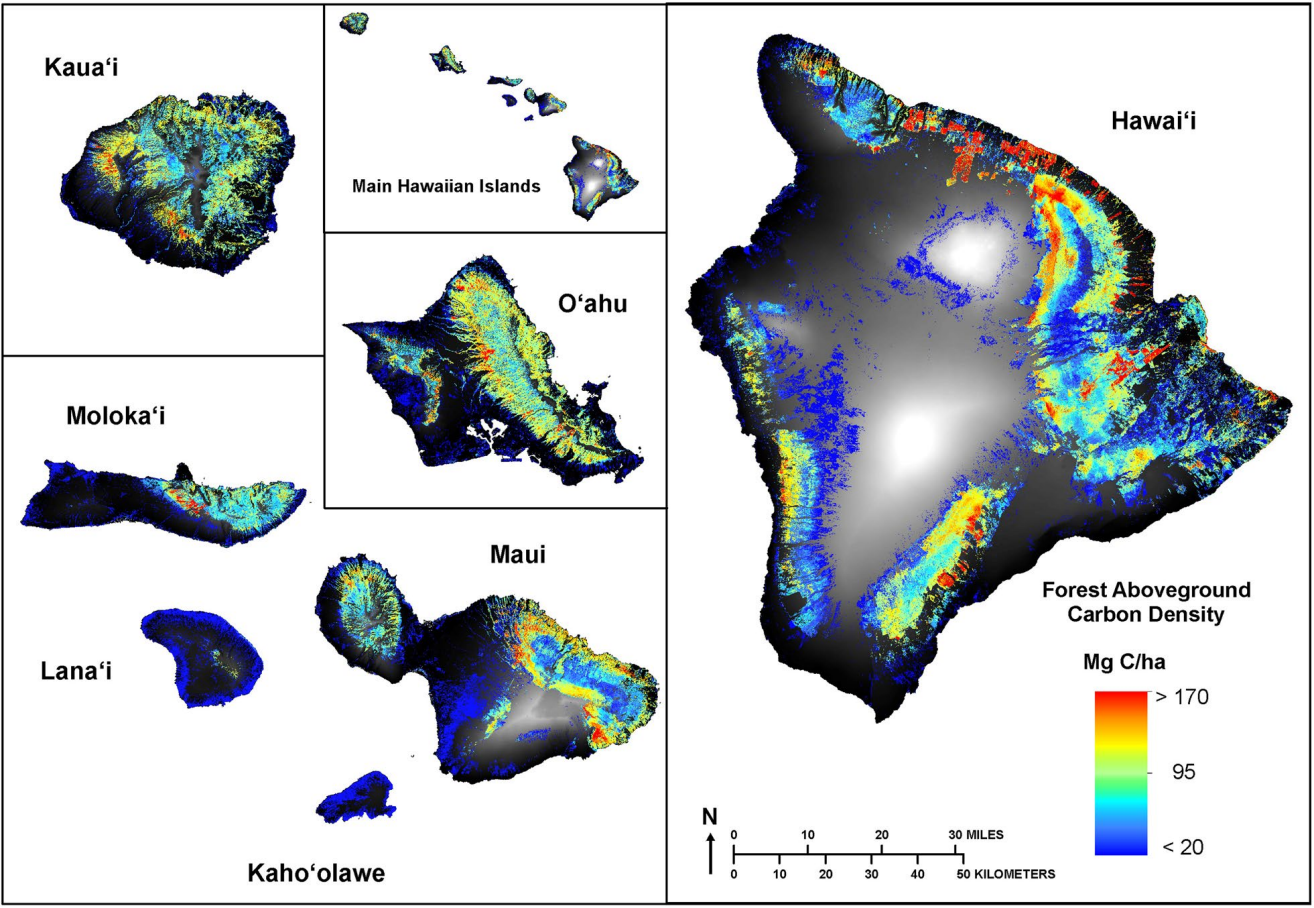
Spatial distribution of forest aboveground carbon density (ACD; Mg C ha^−1^) for the State of Hawaii at 30-m mapping resolution. A map of estimated uncertainty is provided in Additional file 1: Figure S6. The islands are displayed so that their relative sizes are preserved

**Fig. 3:**
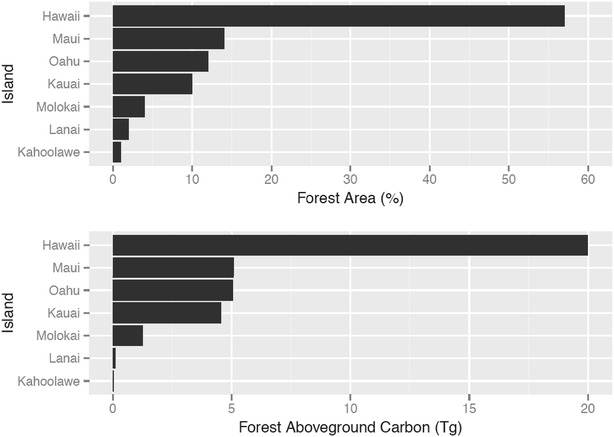
Distribution of forest area and total aboveground carbon stock (Tg = million metric tons) for the main Hawaiian Islands. Percentages are given in terms of the entire State of Hawaii

Total forest cover and aboveground carbon stock for seven main Hawaiian Islands was estimated at 550,065 ha and 36.0 Tg (million metric tons), respectively (Fig. 2; Table 1). A map of estimated uncertainty indicated greatest absolute uncertainties of 20–40 % in very high-biomass forests, with much lower uncertainties in low-to-moderate biomass conditions (Additional file 1: Figure S6). Forest ACD varied widely by island (Fig. 3). Hawaii Island contained 57 % of the total forest cover of the State, and almost 20 Tg of the State’s forest carbon. Kauai, Maui and Oahu islands collectively accounted for 36 % of the total forest cover and 14.7 Tg of aboveground C. Molokai, Lanai, and Kahoolawe together accounted for only 7 % of the State’s forest cover and less than 1.4 Tg C. The small northwest-most island of Niihau was not considered in this study.

**Table 1:** Forest cover and aboveground carbon stock and density for each island and the State’s Districts

Island	Counties and districts	Forest cover (ha)	Aboveground carbon density (Mg C ha^−1^)	Aboveground carbon stock (Tg C)
Hawaii		311,977.0	64.0 + 43.7	20.0
	*Hawaii County*			
	Hamakua	23,391.8	51.4 + 47.4	1.2
	Kau	63,204.2	67.0 + 43.3	4.2
	North Hilo	18,598.8	93.3 + 49.3	1.7
	South Hilo	67,056.8	72.8 + 41.7	4.9
	North Kohala	8341.1	65.9 + 47.9	0.6
	South Kohala	7057.3	47.7 + 31.9	0.3
	North Kona	28,391.2	30.0 + 35.7	0.9
	South Kona	30,635.5	60.8 + 40.8	1.9
	Puna	65,302.4	66.3 + 36.8	4.3
Maui		75,532.9	67.1 + 47.0	5.1
	*Maui County*			
	Hana	29,763.6	78.7 + 41.4	2.3
	Lahaina	22,113.8	33.5 + 40.7	0.7
	Makawao	34,542.2	47.5 + 52.7	1.6
	Wailuku	7543.5	61.1 + 39.5	0.5
	Molokai	23,018.2	54.9 + 37.3	1.3
Molokai		23,018.2	54.9 + 37.3	1.3
Lanai		13,048.5	7.6 + 15.1	0.1
Kahoolawe		5391.1	3.0 + 2.5	0.02
Oahu		64,673.4	78.3 + 40.2	5.1
	*Honolulu County*			
	I	3562.3	77.4 + 39.2	0.3
	II	1648.4	92.4 + 33.3	0.2
	III	2803.9	63.4 + 47.0	0.2
	IV	6669.2	87.9 + 32.9	0.6
	V	10,988.6	95.5 + 32.0	1.1
	VI	14,449.8	80.2 + 38.9	1.2
	VII	4812.6	85.3 + 36.3	0.4
	VIII	5047.9	34.3 + 37.3	0.2
	IX	14,407.3	74.0 + 38.8	1.1
Kauai		56,424.0	80.4 + 35.5	4.5
	*Kauai County*			
	Hanalei	16,033.8	82.8 + 32.0	1.3
	Kawaihau	9020.1	83.0 + 32.1	0.8
	Koloa	3024.7	78.5 + 43.2	0.2
	Lihue	8886.3	79.2 + 32.7	0.7
	Waimea	19,449.3	78.1 + 39.2	1.5

The highest forest ACDs were found on Hawaii Island, reaching 537 Mg C ha^−1^. Maui supported the next highest ACDs, reaching 294 Mg C ha^−1^. We also found extremely variable C stocks on each island (Additional file 1: Figures S7-S10). Aboveground forest C density varied up to three fold among State Districts, which are the minimum State-level political units of civil governance (Table 1). On Hawaii Island, for example, forest ACD values varied from means of 30–93 Mg C ha^−1^ across Districts, yet within Districts, spatial variation in forest ACD ranged from 50 to 111 % of their District means. Moreover, three of nine Districts on Hawaii Island contained two-thirds of the entire island’s forest C stock. The island with the most variable inter-District forest C stocks was Maui.

### Model comparison to FIA plots

**Fig. 4:**
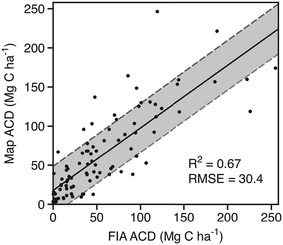
Comparison of Hawaii statewide map of forest aboveground carbon density (ACD) against plot inventory-based estimates of ACD from the US Forest Service FIA plot-inventory data

Comparison of modeled ACD to values estimated from FIA plot inventory indicated good precision (R^2^ = 0.67) and accuracy (average root mean squared error or RMSE = 30.4 Mg C ha^−1^) (Fig. 4). Bias was just 11.2 Mg C ha^−1^, and heteroscedasticity was similar to that derived in plot-inventory comparison studies [27]. These map performances were particularly strong relative to the accuracy of the equation used for estimating ACD from canopy height (Additional file 1: Figure S5).

Here we note the challenges involved in comparing the FIA plot data to mapped C densities based on remote sensing. First, there was an offset of about 6 years between the time the LiDAR flights were completed and the time the FIA measurements were taken in the field. Second, the FIA data in Hawaii were geo-located using non-differentially corrected global positioning system (GPS) instruments. This leads to plot location uncertainties of up to 30 m. The combination of relatively small size (18 m radius), circular shape, and non-contiguity of the FIA plots (see “Methods”), explains higher uncertainty when comparing to ACD estimates in 30 m × 30 m mapping cells. Asner et al. [28] found that mismatches in location and plot shape alone account for up to 15 % uncertainty in field validation studies. Additionally, the allometric scaling applied to the FIA field measurements can result in additional uncertainties of up to 50 % of the plot mean value [29, 30].

Given these, and other sources of uncertainty, we contend that the verification step undertaken here was successful in validating the map results. Nonetheless, validation with FIA or other plots could be significantly improved by more accurate GPS measurements of plot locations, and by employing plot and sampling design that is better suited to validating remotely-sensed estimates of ACD. Specifically, plots should be similar in area to the final grid size and all trees >5 cm dbh should have height and diameter measured in each plot. Better allometry would also decrease uncertainty. Currently, we employ species-specific allometric equations only for the two most dominant native woody tree species (*Metrosideros polymorpha* and *Acacia koa*) and for four non-native tree species. Aboveground biomass for the remaining 114 tree species encountered in FIA plots was estimated using a general model for tropical trees that incorporates diameter, height and wood density [31]. Species-specific allometry for large, widespread non-native tree species, such as *Falcataria moluccana*, would almost certainly reduce uncertainty in estimates of their aboveground biomass.

### Factors affecting carbon stocks

**Fig. 5:**
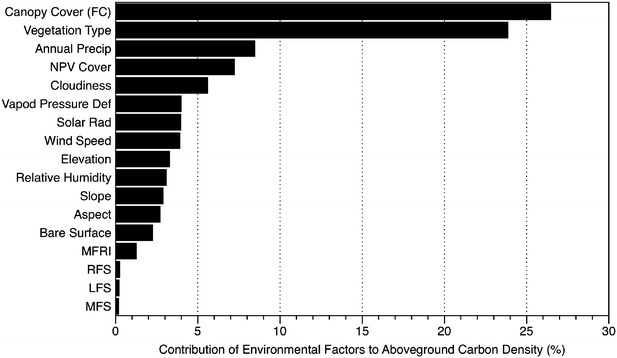
Contribution of each potential explanatory factor determining aboveground carbon density (ACD) in the Hawaiian Islands. Fractional canopy cover (FC), non-photosynthetic vegetation (NPV) cover, and bare surface cover (soils, rock, infrastructure) were derived from sub-30 m resolution Landsat-based satellite mapping of the islands (see [25]). Vegetation type was provided by the Hawaii State GAP vegetation map [34]. *MFRI* mean fire return interval; *RFS* replacement fire severity; *LFS* low fire severity; *MFS* mixed fire severity

The geospatial analysis indicated that fractional canopy cover (FC) was the principal driver of spatial variation in forest carbon stocks throughout the Hawaiian archipelago, accounting for 27 % of the total variance in ACD (Fig. 5). Forest cover was closely followed by forest type, as defined using the vegetation-cover classification, which accounted for an additional 24 % of variation in ACD. Other important factors included mean annual precipitation, vegetation structure, and cloudiness, which individually explained 6–8 % of the ACD variation throughout the islands. Finally, fire return factors, elevation and additional climate variables individually explained 1–4 % of the variability in carbon density.

Note that while the results presented in Fig. 5 account for co-variation in explanatory factors, many of them are ecologically and/or geospatially convolved with one another. For example, forest FC is broadly related to elevation and topographic aspect, with less forest cover often observed at high elevations and on leeward aspects, although low forest FC was also observed in deforested zones at lower elevations on windward aspects. Thus the factor rankings presented here indicate an additional effect of elevation and aspect not already explained by FC alone. Similar inter-factor co-variances occur among the model rankings in Fig. 5. Nonetheless, it is clear that FC and vegetation type explain much of the geographic variation in forest carbon stocks.

### Effects of biological invasion on forest carbon

Although this study is limited to a single time step, the current Hawaii vegetation map allowed us to conduct the first statewide assessment of the large-scale effects of alien plant species on forest C stocks. Numerous plot- to landscape-scale studies have reported on this issue, with highly variable outcomes ranging from no effect of invasion on carbon densities, to increases and decreases in ACD following invasion [17, 23, 28, 32, 33]. Such wide-ranging results stem from underlying variability in the mediating factors, such as time-since-introduction, rates of invasion, relative changes in plant functional and structure types, and environmental filters such as soils and climate. There is thus a general need for large-scale, high-resolution assessments that go beyond local contextual results.

The Hawaii State vegetation map was generated using manual and automated classification of Landsat imagery against aerial photography [34]. Experience with this map in field studies indicates that the “alien-dominated” classes are comprised of mature stands of non-native species, while “native-dominated” classes are comprised of mature stands of native species, particularly dominated by the keystone canopy species *Metrosideros polymorpha* and *Acacia koa*. We focused our analysis on these two groups because the Hawaii State vegetation map alone does not provide sufficient detail to partition the mapped C results into finer levels of invasion, particularly since the invasion process is ongoing and highly dynamic (in favor of alien invasive species dominance). We further partitioned the native- and alien-dominated groups by three major environmental filters known to mediate C stocks: annual precipitation, elevation and substrate age (from volcanic activity dating back to the early Pliocene) (Additional file 1: Table S1).

**Fig. 6:**
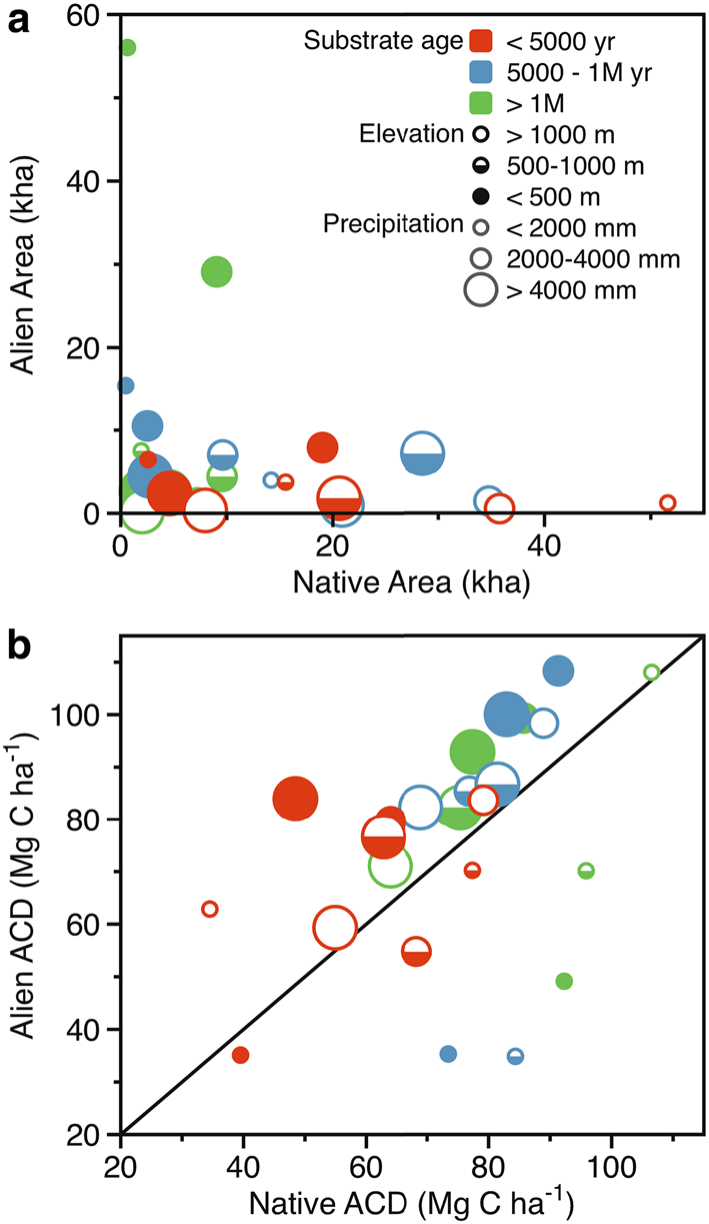
Distribution of **a** forest area and **b** forest aboveground carbon density (ACD) for native-dominated and alien-dominated forests throughout the State of Hawaii. The forests are reported here using the Hawaii State GAP Vegetation map [34] partitioned by lava substrate age, elevation and mean annual precipitation

Our results show that, on medium-to-older substrates in both drier and wetter conditions, the total area of alien-dominated forest exceeds that of native-dominated forest in lower-elevation zones (Fig. 6a). In contrast, the majority of wetter, higher-elevation and/or older-substrate conditions remain dominated by native forest cover. Critically, however, we found that ACD is greater in native-dominated forests in low-to-medium elevation, dry-to-mesic regions of the islands, whereas alien-dominated forests tend to have slightly higher ACD levels in wetter environments across the board (Fig. 6b). At these broad multi-island scales, substrate age played only a small role in determining the *relative* difference in alien- and native-dominated forest ACD. This suggests strong limiting effects of nutrient-poor soils on growth and biomass accumulation for all species, independent of origin [35]. In contrast, higher biomass of native forest canopies in drier zones on older substrates may reflect evolutionary adaptation to these environments, as well as a lack of analog tree taxa in the current alien species pool on the islands.

Our results are also suggestive of how native biological diversity intersects with C storage, and how alien invasive species alter those relationships. For example, higher-elevation, drier forests on older substrates may be dominated by alien forest cover (smallest solid green dot; Fig. 6a), but native-dominated forests in similar environments support twice the stored C on a per-area basis (Fig. 6b). Thus actions to conserve and restore high-elevation native ecosystems yield a co-benefit of increased C storage. On the other hand, higher-elevation, drier conditions on younger substrates are areas currently dominated by native forest cover (open small red dot; Fig. 6a), but alien species can double the ACD levels in these environments (Fig. 6b). Forest managers and conservationists can use these landscape-scale relationships as trade-offs in planning efforts to increase C storage while managing for biological diversity [36, 37].

### Forest carbon protections and opportunities

**Fig. 7:**
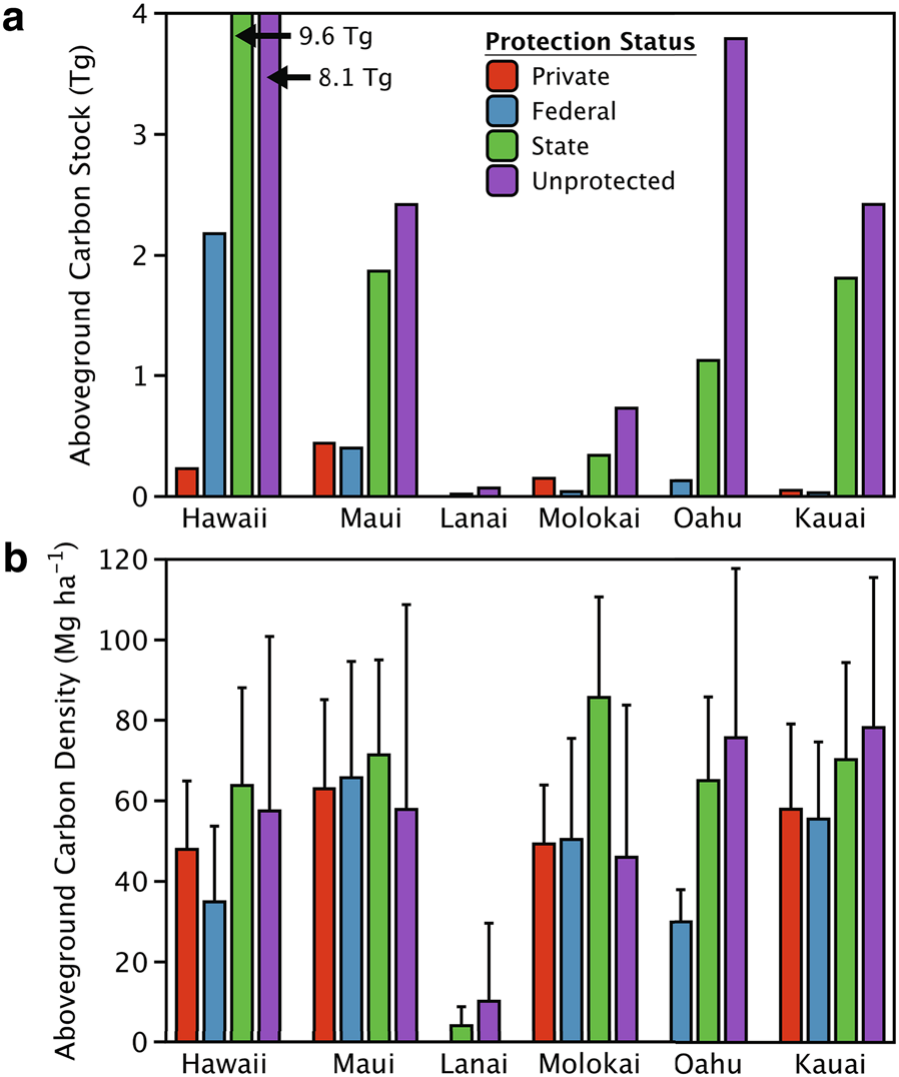
Distribution of forest **a** aboveground carbon stock and **b** aboveground carbon density on protective reserves managed by State, federal and private organizations, as well as unprotected forested lands

High-resolution C mapping also affords a way to assess current protections, threats and opportunities for sequestered carbon and generating healthy forests via land-use allocation and management [21]. Using land tenure data provided by the State of Hawaii, we quantified C stocks and densities on State, federal and private reserves. Of the total aboveground forest C stock found on the islands (36 Tg C), about 18.5 Tg C or 51 % is officially protected on State (e.g., Natural Area Reserves; Forest Reserves), federal (National Parks; Wildlife Refuges) and private (The Nature Conservancy; Kamehameha Schools lands) lands covering 257,691 ha (Fig. 7a, Additional file 1: Table S2). This is almost equally matched by forests outside of protected reserves, which in total cover more land area at 292,374 ha, but which contain 17.5 Tg of aboveground C. This finding indicates that a large amount of forest C could be incorporated into more formal reserve protections. Moreover, we found that reserve ACD averages 61.8 ± 22.3 Mg C ha^−1^, whereas non-reserve forests have carbon densities of 59.6 ± 34.2 3 Mg C ha^−1^ (Fig. 7b). Combined, these results underscore the C-storage benefit of adding long-term protection status to remaining island forests; Total forest aboveground C stock increases linearly with increasing reserve area (Additional file 1: Figure S11).

On all islands, 189 State-managed reserves hold the vast majority of protected carbon stocks—14.8 Tg C, while 25 federal and 14 private reserves contain just 2.8 and 0.9 Tg C, respectively (Additional file 1: Table S2). Carbon densities are highest in State reserves (66.3 ± 23.2 Mg C ha^−1^), followed by private (56.1 ± 19.3 Mg C ha^−1^) and federal reserves (41.4 ± 17.6 Mg C ha^−1^). Differences in forest carbon densities are reflective of the location of the reserves (lowland vs. montane, wet windward vs. dry leeward) as well as species composition and management. A desired outcome of this work is to provide forest managers and the public with information to compare, for example, carbon stocks on a reserve-by-reserve basis against environmental maps, to identify opportunities for increasing C densities through conservation and management actions.

### Replication on oceanic island chains

The approach we have developed and tested here for high-resolution mapping of aboveground forest carbon density is intended for replication on oceanic islands worldwide, but also any set of highly heterogeneous landscapes. The methodology is based on a previously established strategy that relies on airborne LiDAR sampling of forests found across a range of ecological conditions, but limited to one island [23]. Here we greatly advanced the approach by extending the initial LiDAR sampling of a single island, via a machine-learning algorithm [38, 39], to the multi-island or archipelago scale using a suite of environmental maps and satellite data that is, in combination, sufficiently sensitive to variation in the LiDAR-based estimates of canopy height. Shared environmental characteristics among neighboring islands usually include geology, climatic zones, and dominant vegetation types. Satellite-based metrics of forest structure, derived from Landsat-based spectral mixture analysis, are time-variant and key to the linkage with the LiDAR data. Strategically, these Landsat-based metrics can be updated through time using the fully automated CLASlite software [25].

The conversion of either LiDAR-scale or modeled canopy height to estimates of ACD requires plot-aggregate allometric equations [40]. This worked well in Hawaii, relative to plot-estimated ACD from U.S. Forest Service inventory data. The universal or regional plot-aggregate allometric equations proposed by Asner et al. [40] have also worked reasonably well in other regions [17, 41, 42], and they tend to result in mismatches between LiDAR-based and field-based estimates of ACD of 10–15 % when applied at 1-ha spatial resolution [26]. Nonetheless, application of these conversion equations to oceanic islands requires further validation, particularly for isolated islands in which vegetation types (and thus allometrics) may diverge from general databases.

There is an initial cost for installing a forest C monitoring program on any given island chain or archipelago. It includes an initial airborne LiDAR survey of one island or part of the archipelago, which varies widely in cost depending upon whether the data are sourced from non-profit, government, or commercial organizations. Our LiDAR data collection and processing cost was approximately $150,000 for the Island of Hawaii, but costs have greatly declined since the data acquisition was made for this study [43]. The LiDAR component was followed by personnel and computing costs required to link the LiDAR data to the satellite imagery and for validation work. However, the satellite imagery was free of charge, and CLASlite is also currently available at no charge [44], thereby providing us with a low-cost way to complete the initial carbon map. Moreover, the free imagery and software makes updates to the map extremely cost-efficient, likely requiring the effort of a single geospatial technician for the State of Hawaii. Even if field inventories could be done at large geographic scales on a spatially contiguous basis, which is not possible, the recurring costs would be extremely high for each monitoring step through time.

## Conclusions

We have shown that a combination of one-time airborne LiDAR data acquisition, and freely available satellite data with automated analysis, can provide effective forest C mapping and monitoring of oceanic islands. The method is highly replicable and cost-effective. From the first map generated, and with regular updates using satellite data over time, assessments of C storage can be derived by political entity (e.g., State Districts), land-use allocation (e.g., protected vs. unprotected areas), or any other unit of governance or management. Moreover, changes in C stocks and densities can be continually assessed in the face of rapid environmental changes, such as climate, fire and biological invasion. The resulting information is spatially explicit, allowing for actions that promote sustainability of forests and the services they provide to island biodiversity and societies. High-resolution monitoring approaches also provide a geography of forest C stock that facilitates the inclusion of multiple stakeholders ranging from individual landowners to national governments. The resulting empowerment afforded by this type of ecological information will be important to the protection, enhancement and/or restoration of island ecosystems in the future.

## Methods

Our mapping approach is summarized in Fig. 1. The necessary technologies are airborne Light Detection and Ranging (LiDAR), which yields highly detailed measurements of forest canopy height and vertical canopy profile, and satellite-derived maps of environmental variables and forest canopy fractional cover. A second component relies on machine learning algorithms to scale airborne LiDAR samples of one island up to multi-island or archipelago maps. Several studies have employed a Random Forest Machine Learning (RFML) algorithm to model the relationship between LiDAR-based estimates of forest structure or biomass and a suite of satellite data sets [19, 21, 45, 46]. RFML fits multiple environmental datasets (predictors) to estimates of vegetation structure or biomass (response), as described later. In doing so, a direct scaling of LiDAR samples to full-coverage maps can be derived without artificial boundaries between ecosystems that often occur using traditional stratification approaches.

Random Forest Machine Learning also provides quantitative information on which predictors (e.g., satellite data) are most important in determining the response variable (LiDAR-derived canopy height) [47]. Here the importance of a predictor to the RFML model was assessed by randomly permuting the values of the factor within a validation dataset, and processing the validation data through the regression trees. In our implementation of RFML, a temporary validation dataset is created to build each regression tree, and is chosen as a randomly selected set of 250 samples left out of the full training dataset. To assess the importance of a single factor, we compared the mean square error (MSE) values of the validation data both before (MSE_i_) and after (MSE*_i_) randomly permuting the values of the factor for each tree [48]. For each tree i, the difference between MSE_i_ and MSE*_i_, divided by MSE_i_, was collected. The importance of the given factor was then taken to be the mean of these relative difference values across all trees. By repeating the above procedure for each explanatory factor, the relative importance of each factor could be compared.

### LiDAR data acquisition and analysis

LiDAR data were collected using the Carnegie Airborne Observatory [49]. Flights covered 379,337 ha of Hawaii Island (Additional file 1: Figure S1) including all major forest types (Additional file 1: Figure S2b) [23]. LiDAR data were collected at 1000 or 2000 m above ground level, using two corresponding configurations: higher resolution with 0.56 m on-the-ground laser spot spacing, 24° field of view (FOV) and a 70 kHz pulse repetition frequency; low resolution with a 1.12 m spot spacing, 30° FOV and a 50 kHz pulse repetition frequency, respectively. Ground cover was sampled along parallel flight lines with 50 % overlap to ensure LiDAR coverage of no less than 4 laser shots m^−2^.

Mean top-of-canopy height (TCH) was calculated for each 30 m × 30 m grid cell of LiDAR coverage on Hawaii Island (Additional file 1: Figure S1). To create this layer, the laser range measurements from the LiDAR were combined with the embedded high resolution Global Positioning System-Inertial Measurement Unit (GPS-IMU) data to determine the 3-D locations of the laser returns. This calculation produced a ‘cloud’ of LiDAR data. The LiDAR data cloud was processed to identify where the laser pulses penetrated the canopy volume, reaching the ground surface, from which a digital terrain model (DTM) was produced. This was achieved using a 10 m × 10 m filter kernel throughout the LiDAR coverage, and the lowest elevation in each kernel was deemed as possible ground detection. These filtered points were then evaluated by fitting a horizontal plane through each point. If the closest unclassified point was <1.5 m higher in elevation, the pre-filtered point was finalized as a ground-classified surface point. This process was repeated until all potential ground points within the LiDAR coverage were evaluated. A digital surface model (DSM), which is essentially the top-most surface (e.g., canopies, buildings, exposed ground), was also generated based on interpolations of all first-return points at 1.12 m spatial resolution. The DTM and DSM were combined as a tightly matched pair of data layers. The vertical difference between them resulted in a model of top-of-canopy height (TCH) at 1.12 m spatial resolution throughout the 379,337 ha LiDAR sampling coverage. Validation studies of this CAO LiDAR TCH estimation approach have shown it to be highly accurate across a wide range of forests including extremely densely foliated, tall tropical forests exceeding 60 m in height [28, 42].

### Environmental predictor variables

We used 17 environmental predictor variables from co-aligned spatial datasets covering State of Hawaii to model canopy height based on the LiDAR TCH measurements made on Hawaii Island (Additional file 1: Figure S2-S4). All predictor variables were gridded at 30-m spatial resolution. Three predictor variables were fractional cover of forest canopy (FC), non-photosynthetic vegetation (NPV), and bare surfaces. These were determined from nine primary Landsat-8 images collected in 2013 and 2014. The mosaic of nine images included a few small cloud-covered areas, so those areas were backfilled with Landsat-7 and Landsat-8 data going back to 2010. The Landsat mosaic was run through a probabilistic spectral mixture analysis algorithm embedded in the CLASlite forest monitoring software package [25]. These fractional cover images have been validated and used in numerous studies in Hawaii and elsewhere [e.g., 23, 50].

An important additional predictor variable was the Hawaii State GAP vegetation map, which provides the highest resolution and most widely used vegetation cover type information for the State of Hawaii. The version used was based on Gon et al. [34], with improvements based on high-resolution satellite images and other more recent vegetation mapping information [51]. Three additional predictor variables were derived from 30-m Shuttle Radar Topography Mapping (SRTM) mission data: elevation, slope and aspect. In addition, mean annual precipitation (MAP), mean wind speed at 2 m above ground, vapor pressure deficit, total solar radiation, mean relative humidity, and cloud frequency data were acquired from http://climate.geography. http://hawaii.edu/downloads.html. Finally, we used four fire-related predictor variables: low fire severity (LFS), mixed fire severity (MFS), replacement fire severity (RFS), and mean fire return interval (MFRI) provide by http://www.landfire.gov/fireregime.php.

These 17 predictor maps and the 30-m LIDAR-derived TCH map were applied to the RFML model for Hawaii Island to develop the prediction-based regression trees. The regression trees were then used to predict TCH values across the entire State of Hawaii using the 17 predictor maps as input.

### Estimating aboveground carbon density

We estimated ACD from the statewide TCH map using a plot-aggregate allometric scaling approach [26]. A biophysical link was previously developed to quantitatively link mapped TCH to field estimates of ACD by applying regional plot-aggregated estimates of vegetation wood density and diameter-to-height relationships. To develop a TCH-to-ACD calibration for Hawaiian forests and other vegetation types throughout the State, we used 209 field plots located on Hawaii Island for which ACD was measured using field plot-based inventory measurements as detailed by Asner et al. [23]. The resulting calibration between TCH and ACD is shown in Additional file 1: Figure S5, with in R^2^ = 0.82 and RMSE = 78.7 Mg C ha^−1^. The final calibration equation for relating TCH to ACD was: ACD = 3.744 * TCH^1.391^.

### Uncertainty map

The uncertainty of the mapped ACD estimates was estimated by developing a relationship between the mapped ACD values and the RMSE of ACD for those areas on Hawaii Island covered by the LiDAR data [21]. These RMSE values were partitioned into 30 bins across the range of RFML-modeled ACD values. A polynomial was fit to model the RMSE of an ACD estimate as a function of its predicted ACD value. The polynomial was then applied to the ACD map to produce an estimate of ACD uncertainty (Additional file 1: Figure S6).

### Map validation

To evaluate the accuracy of the final carbon map, we compared data from the map to georeferenced plots surveyed across the Hawaiian Islands in 2011 and 2012 by the United States Department of Agriculture Forest inventory and Analysis (FIA) Program. The FIA Program is a national network of plots designed to represent all forest conditions across the United States [52]. Each FIA plot is a cluster of four circular 7.32-m radius subplots arranged in a fixed pattern. All trees and tree ferns ≥12.7 cm diameter at breast height (dbh; 1.37 m above the ground) had diameter, height, and species recorded in each subplot. Trees and tree ferns <12.7 cm dbh had diameter, height, and species were recorded in microplots, which are 2.07 m radius plots located within each subplot. Macroplots, which are 17.95 m radius and immediately surround each subplot, are usually reserved for destructive sampling. However, FIA plots sampled in Hawaii in 2011–2012 using the ‘experimental forest’ (EXPFOR) protocol (n = 96) had all trees ≥12.7 cm dbh measured in Macroplots as well, greatly enlarging the sample footprint of each plot. We used data from these 96 EXPFOR FIA plots to validate the accuracy of the final carbon map.

We estimated ACD for each tree measured in the 96 FIA plots using a combination of species-specific and general diameter-to-ACD and height-to-diameter models. We used locally derived, species-specific diameter to ACD models for eight species, including the two most common species in the FIA dataset: *Metrosideros polymorpha* and *Acacia koa* (Additional file 1: Table S3). For all other species, and for large trees that exceeded the diameter range of species-specific diameter-to-ACD models, we used a general allometric model for tropical trees developed by Chave et al. [31] that uses diameter, height, and wood density to estimate ACD (Additional file 1: Table S4). When the Chave model was employed, we used species-specific wood density values from Hawaii [23] and a global wood density database [53]. If a species-specific wood density value was unavailable, we used a mean value for the genus, and if this was not available we used a default value of 0.5 (Additional file 1: Table S5). We note here that wood densities are difficult to find for some commonly occurring oceanic island species, and thus we encourage research and measurement in this area. Occasionally, a height measurement was lacking for trees requiring the general Chave model. In these instances, we used locally derived, species-specific diameter to height models from Asner et al. [23]. When no species-specific diameter-to-height model was available, we used a general diameter-to-height model developed by Chave et al. for tropical trees that incorporates an environmental stress *E* parameter. Plot-level ACD was estimated by (1) estimating aboveground biomass (AGB) per unit area of microplots and macroplots within each FIA plot; (2) summing AGB per unit area within each FIA plot (n = 96); and (3) multiplying plot-level AGB per unit area by 0.48 to estimate ACD. The ACD of the 96 FIA plot locations were extracted from the statewide carbon map and averaged in a 3 × 3 pixel window (~1 ha) centered on each plot location.

## Additional file

**Additional file 1.** Supporting figures and tables.

## Abbreviations

ACD: aboveground carbon density
AGB: aboveground biomass
C: carbon
FC: fractional cover
FIA: Forest Inventory and Analysis
LFS: low fire severity
LiDAR: Light Detection and Ranging
MFRI: mean fire return interval
MFS: mixed fire severity
NPV: non-photosynthetic vegetation
PV: photosynthetic vegetation
RFML: Random Forest Machine Learning
RFS: replacement fire severity
TCH: top of canopy height
