# Supplementary material for: Rapid forest carbon assessments of oceanic islands: a case study of the Hawaiian archipelago
Source: Carbon Balance Manag. 2016 Jan 8;11:1. doi: 10.1186/s13021-015-0043-4 (PMC4705141; doi:10.1186/s13021-015-0043-4)
Supplement: Supplementary file 1 — Additional file 1. Supporting figures and tables. [file 13021_2015_43_MOESM1_ESM.pdf]

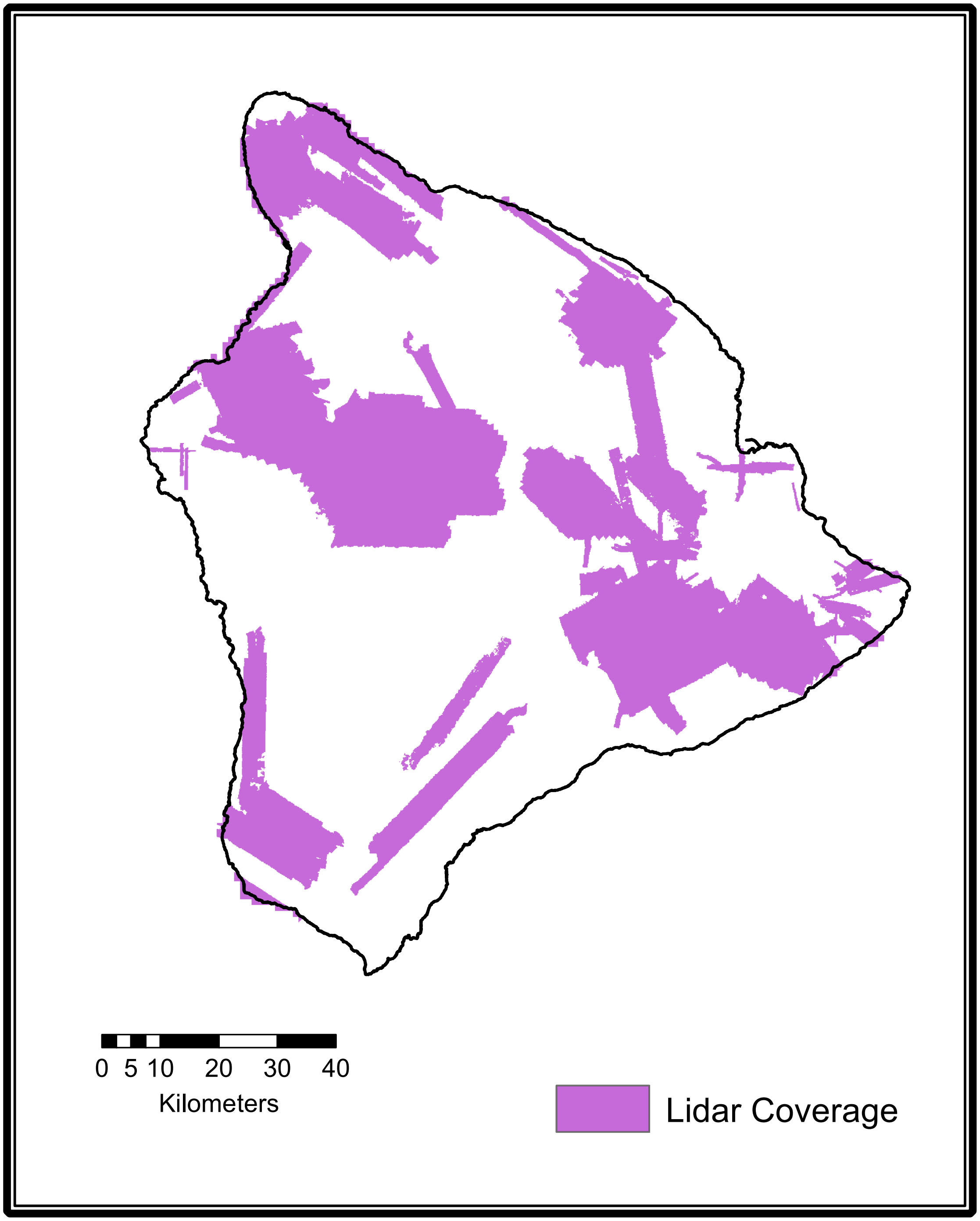

Figure S1. Carnegie Airborne Observatory LiDAR coverage of Hawaii Island.

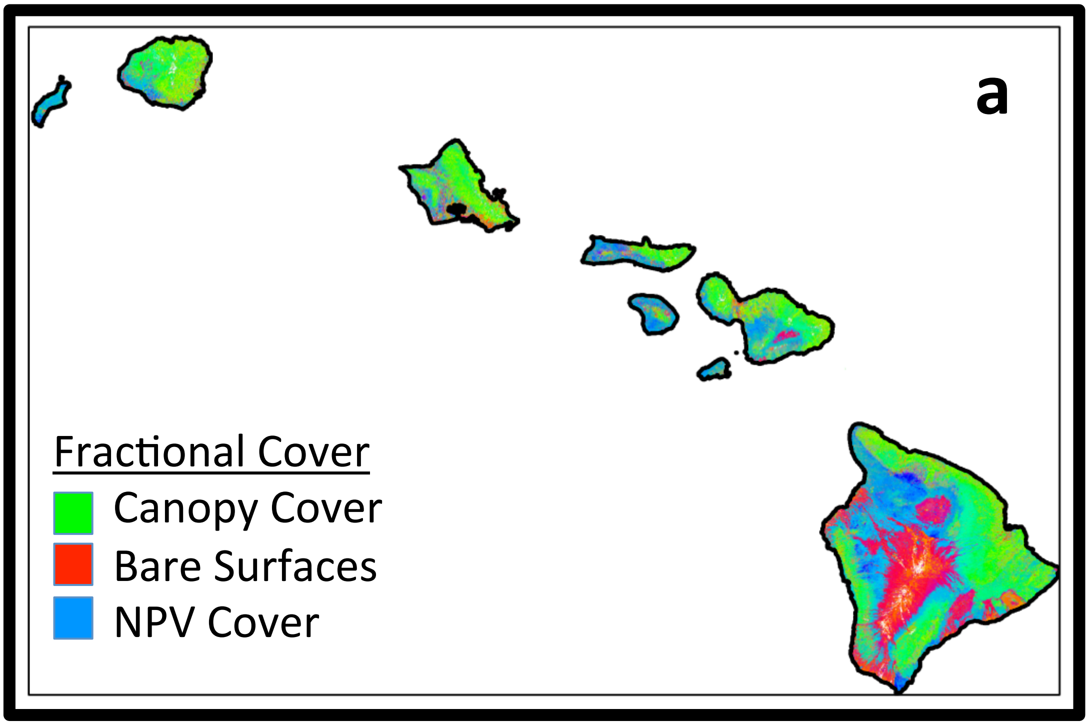

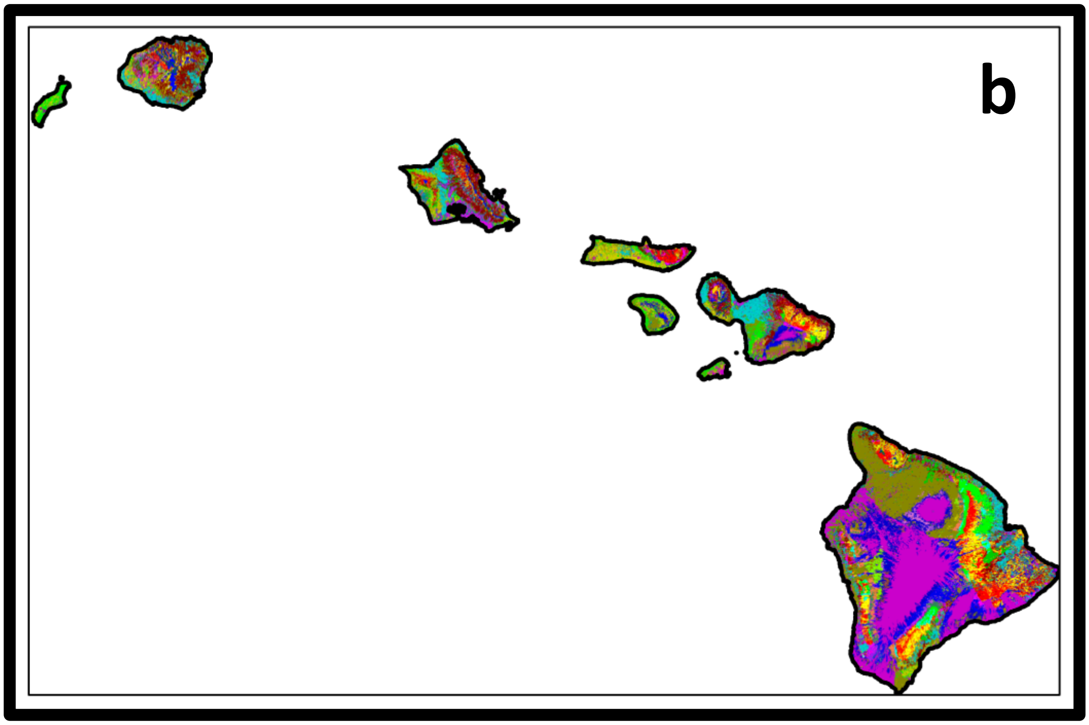

Figure S2. (a) Fraction canopy cover, bare surfaces and non-photosynthetic vegetation (NPV) cover derived from Landsat imagery using the CLASlite forest monitoring software. (b) Hawaii State vegetation map from [27].

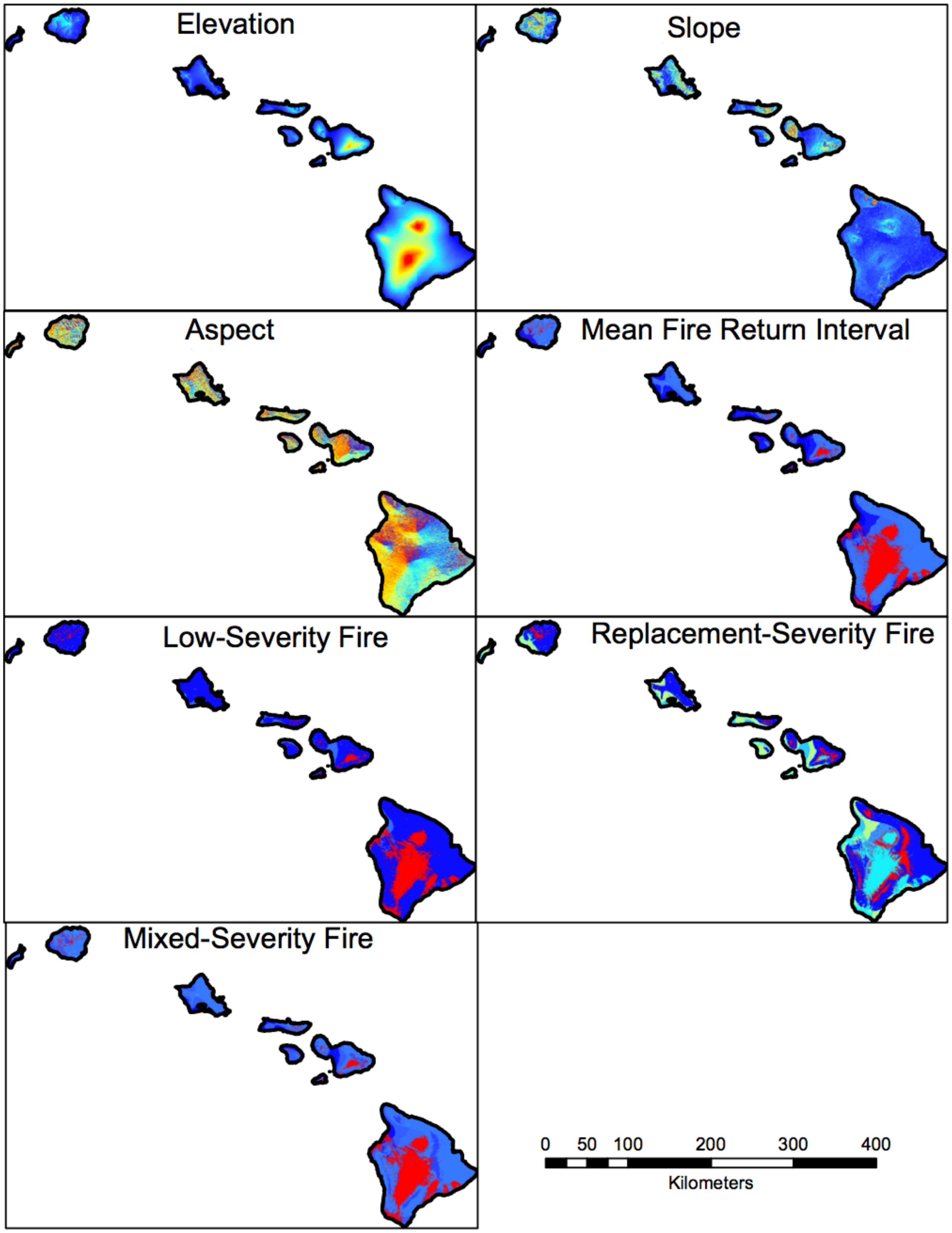

Figure S3. Data layers used for Random Forest Machine Learning (RFML) upscaling of LiDAR-based top-of-canopy height (TCH) data to the State of Hawaii.

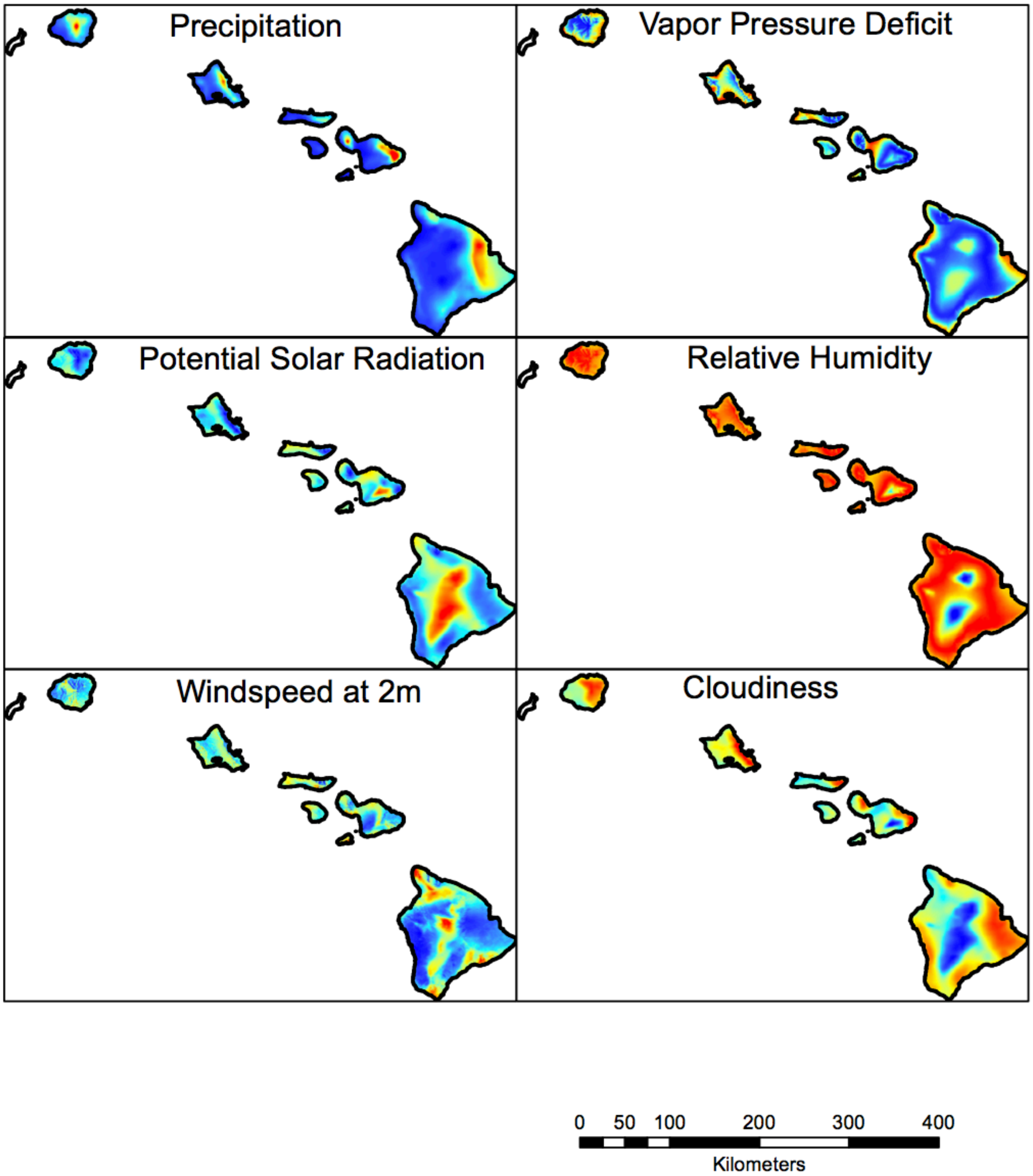

Figure S4. Data layers used for Random Forest Machine Learning (RFML) upscaling of LiDAR-based top-of-canopy height (TCH) data to the State of Hawaii.

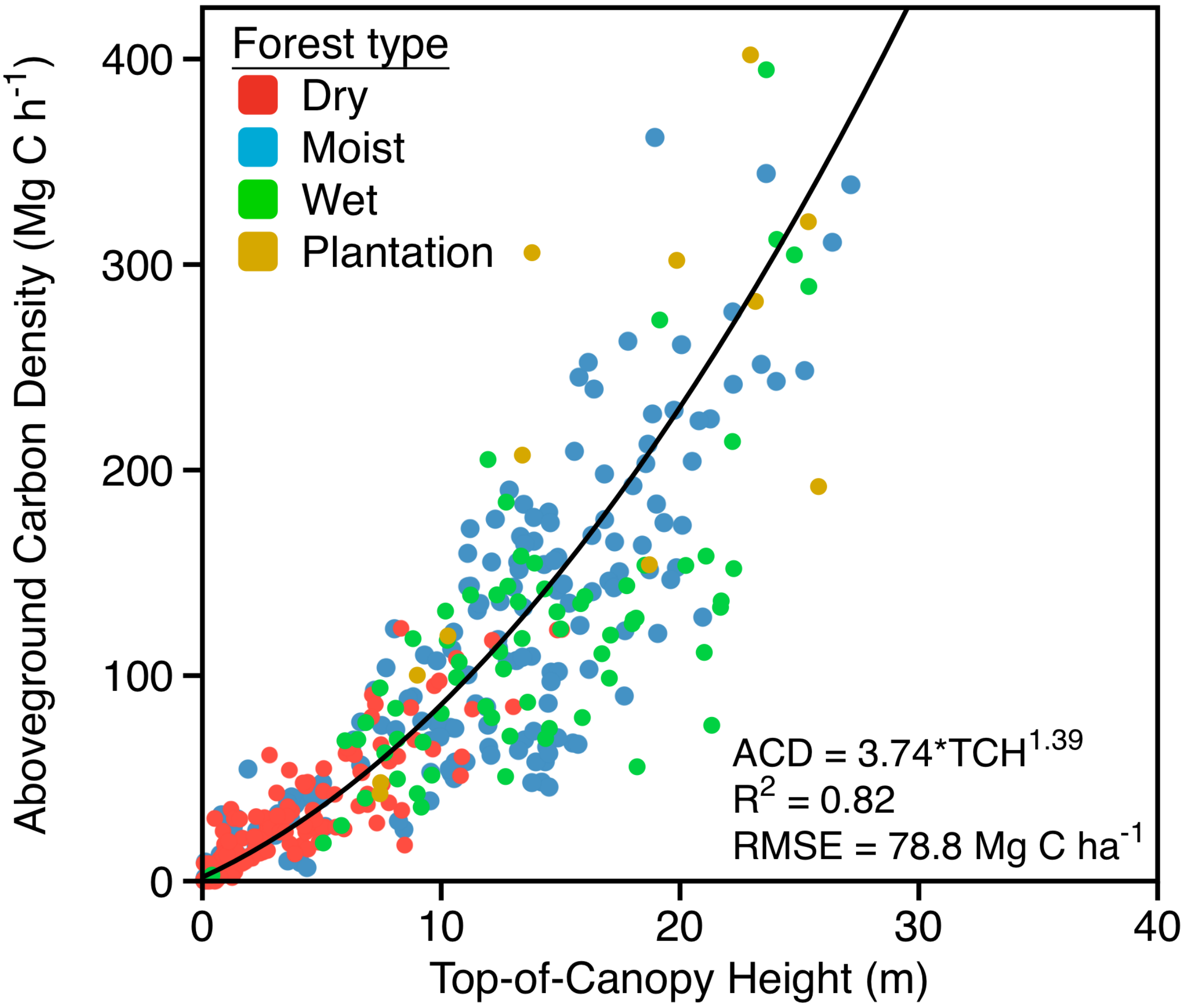

Figure S5. Calibration of top-of-canopy height (TCH) to aboveground carbon density (ACD) using a general plot-aggregate scaling approach from [32]. This equation was used for converting the Statewide model of TCH to ACD at 30-m spatial resolution.

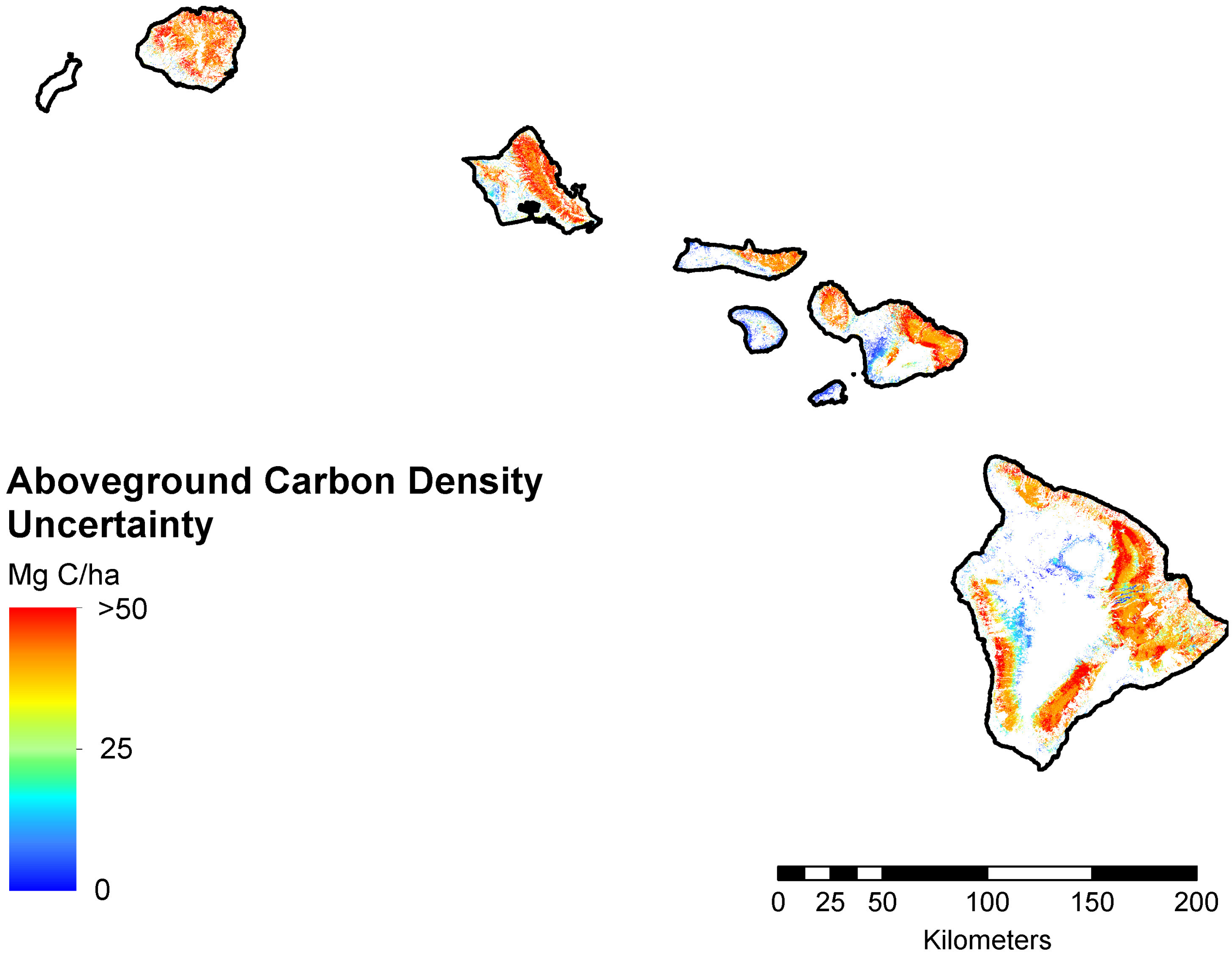

Figure S6. Map of estimated uncertainty in aboveground carbon density (ACD) at 30-m spatial resolution.

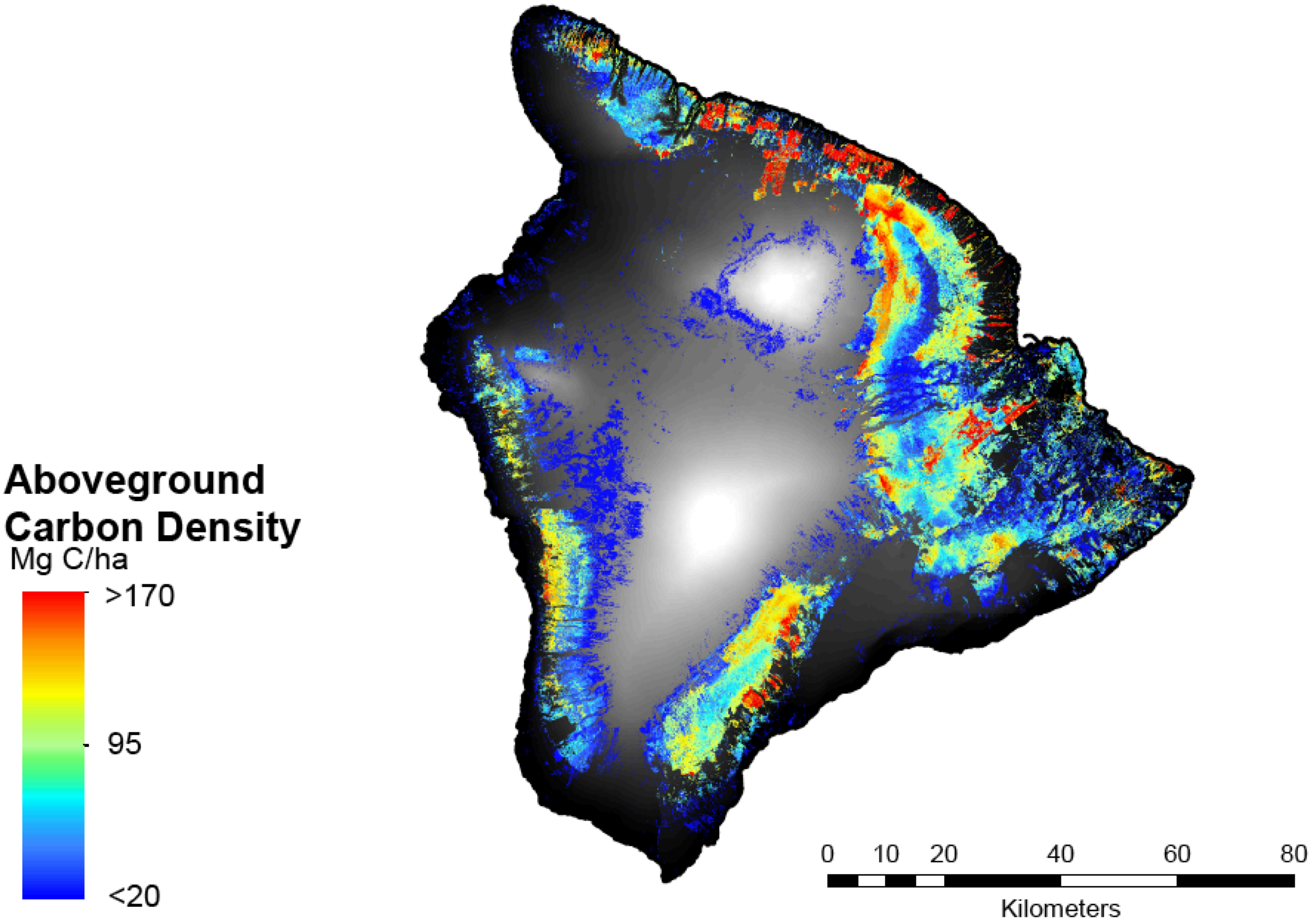

Figure S7. Zoom image of aboveground carbon density (ACD) for Hawaii Island extracted from Figure 2 of main text.

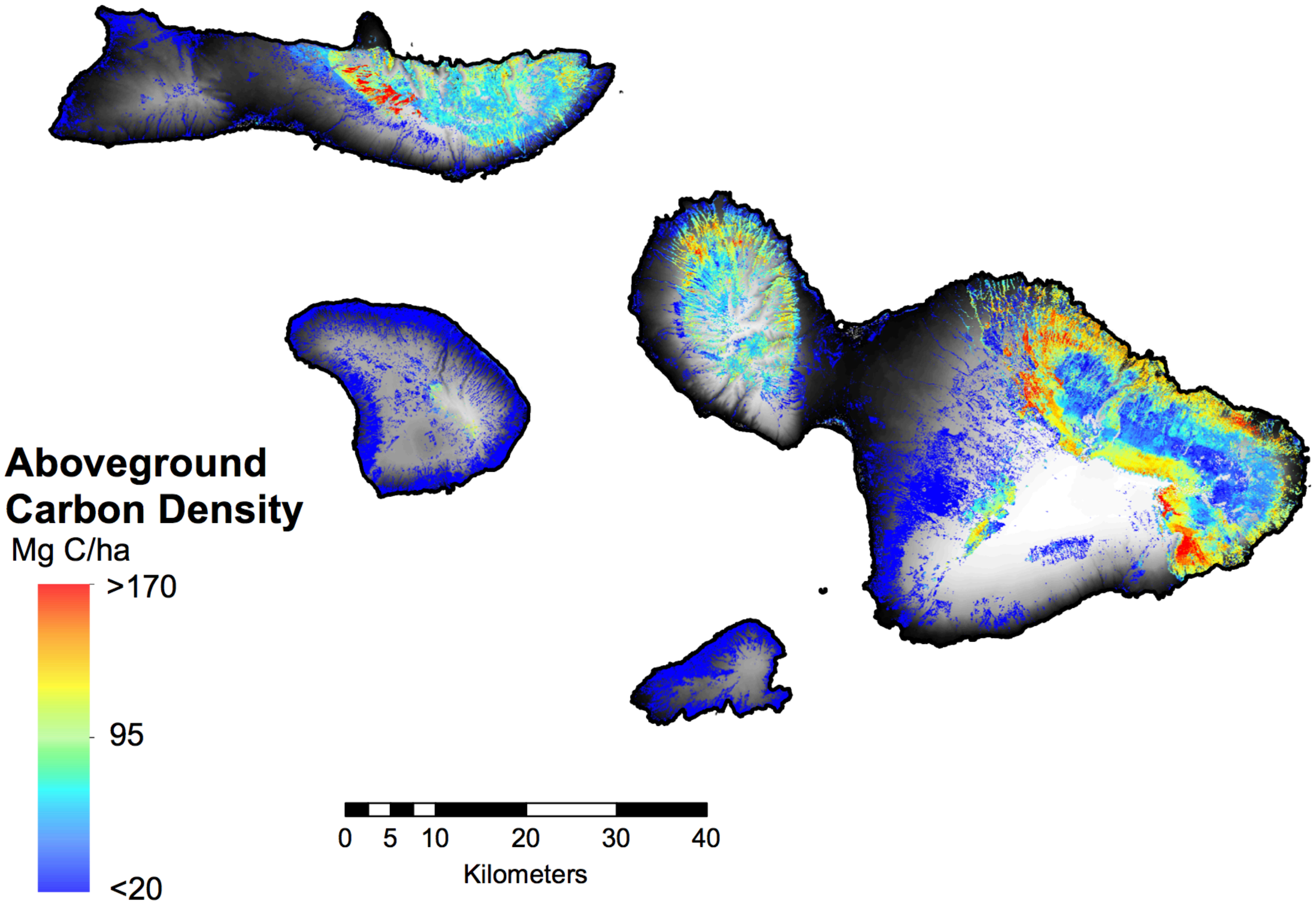

Figure S8. Zoom image of aboveground carbon density (ACD) for the Islands of Molokai, Maui, Lanai, and Kohoolawe extracted from Figure 2 of main text.

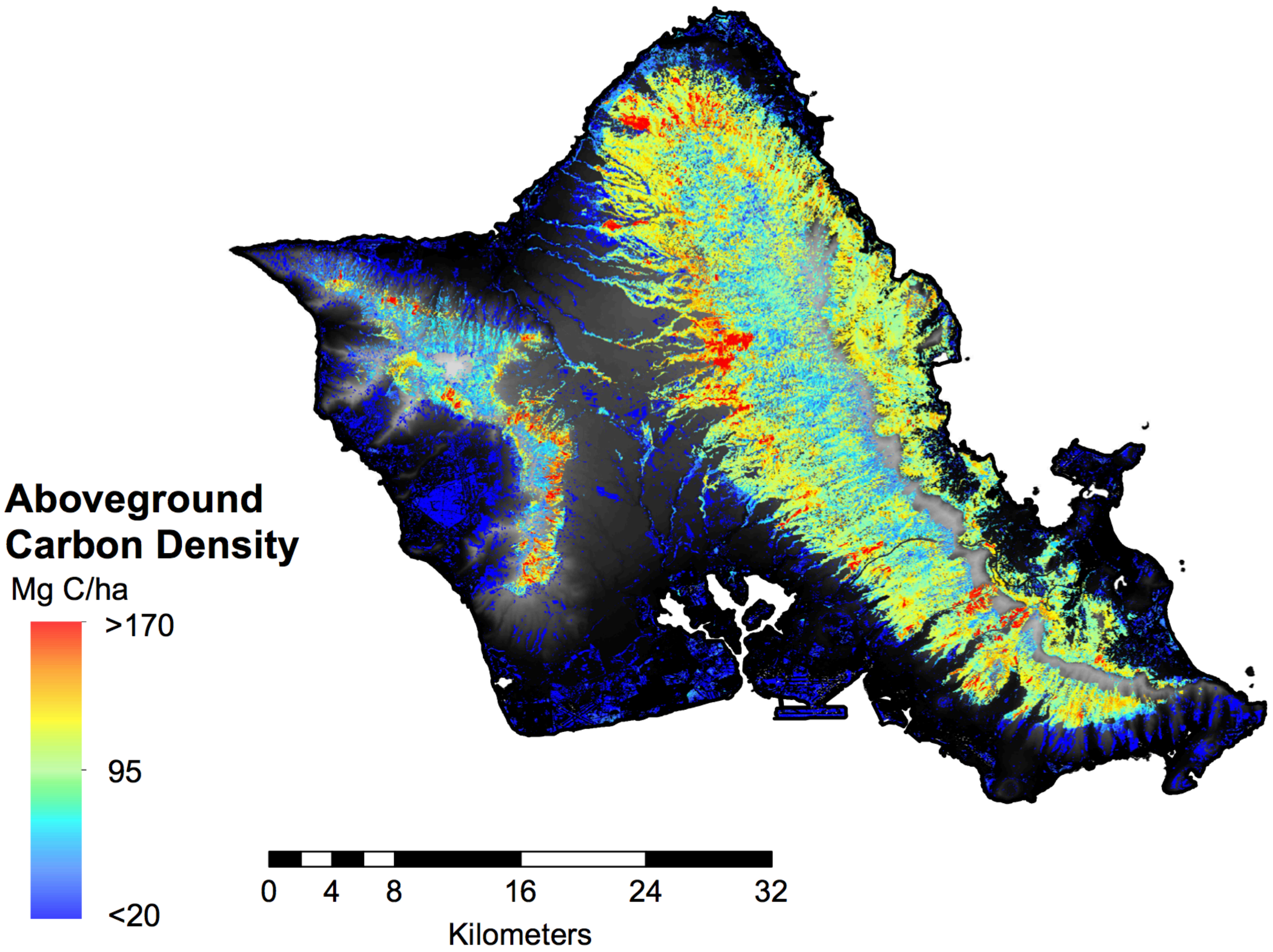

Figure S9. Zoom image of aboveground carbon density (ACD) for Oahu Island extracted from Figure 2 of main text.

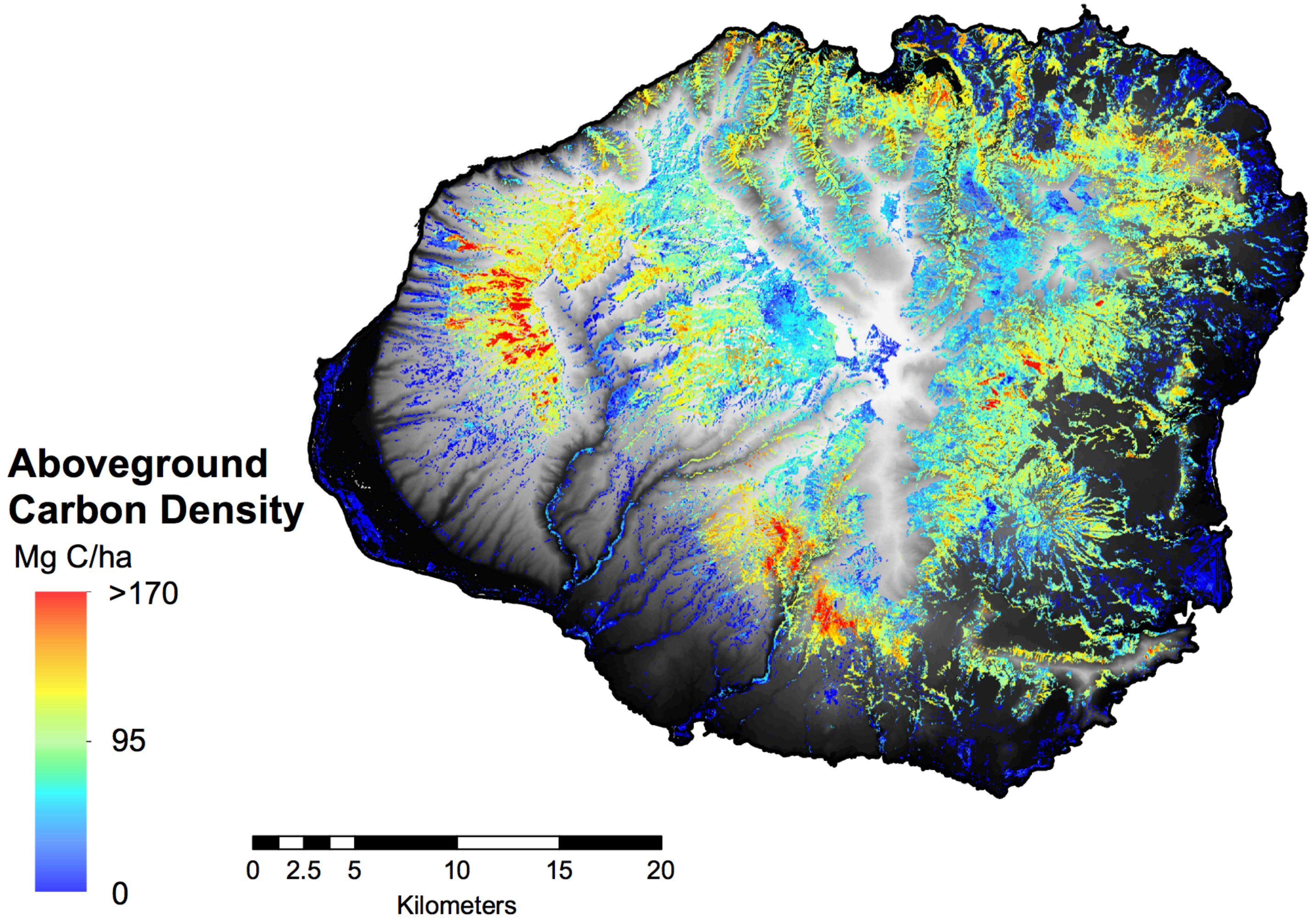

Figure S10. Zoom image of aboveground carbon density (ACD) for Kauai Island extracted from Figure 2 of main text.

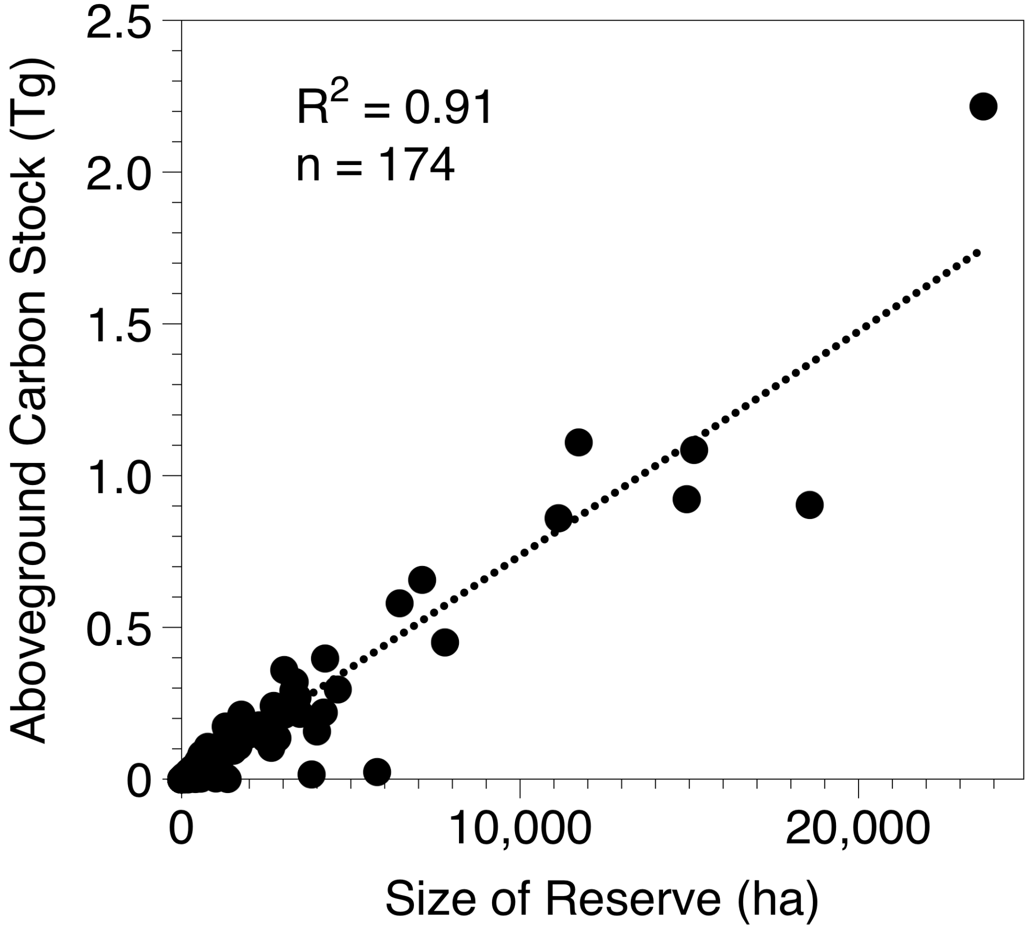

Figure S11. Relationship between reserve size and total aboveground carbon stock for 174 reserves throughout the State of Hawaii.

Table S1. Aboveground carbon densities for alien-dominated and native forests in differing environmental conditions derived from the forest aboveground carbon density (ACD) map of the Hawaiian Islands (Figure 2).

Environment			Native-dominated		Alien-dominated	
Substrate Age	Mean Annual Precipitation	Elevation	Aboveground Carbon Density (Mg C ha^{-1})	Area (ha)	Aboveground Carbon Density (Mg C ha-1)	Area (ha)
> 1M yr	< 2000 mm	< 500 m	92.3+26.8	640	49.1+47.8	56,033
		500-1000 m	95.9+29.2	1,935	70.3+43.7	7,515
		> 1000 m	106.5+27.1	1,158	108.0+26.8	1,238
	2000-4000 mm	< 500 m	85.7+26.1	9,050	99.3+28.6	29,101
		500-1000 m	73.5+21.4	9,561	81.9+28.7	4,428
		> 1000 m	76.8+24.6	7,206	83.6+26.0	1,260
	> 4000 mm	< 500 m	77.4+25.0	1,950	92.9+26.0	2,653
		500-1000 m	75.4+21.2	4,542	82.3+25.6	2,567
		> 1000 m	64.0+20.4	2,018	71.1+23.2	205
5000-1M yr	< 2000 mm	< 500 m	73.4+40.7	470	35.3+46.4	15,429
		500-1000 m	84.4+43.2	1,588	34.8+41.7	4,244
		> 1000 m	18.9+29.7	14,186	61.4+39.7	3,992
	2000-4000 mm	< 500 m	91.3+35.7	2,523	108.3+41.5	10,510
		500-1000 m	76.9+35.3	9,617	85.4+48.3	7,021
		> 1000 m	88.9+42.6	34,767	98.3+57.1	1,439
	> 4000 mm	< 500 m	82.9+31.9	2,791	100.0+33.2	4,502
		500-1000 m	81.4+32.6	28,489	86.7+24.8	7,226
		> 1000 m	68.9+38.7	20,854	82.3+33.4	1,001
< 5000 yr	< 2000 mm	< 500 m	39.5+44.2	2,585	35.1+36.8	6,458
		500-1000 m	77.4+41.0	15,544	70.3+39.6	3,748
		> 1000 m	34.5+33.5	51,602	62.9+32.9	1,209
	2000-4000 mm	< 500 m	64.0+37.8	19,023	79.6+49.0	7,906
		500-1000 m	68.2+37.1	7,919	54.8+33.4	396
		> 1000 m	79.2+41.8	35,767	83.7+38.2	524
	> 4000 mm	< 500 m	48.4+28.1	4,608	83.9+32.1	2,381
		500-1000 m	62.8+34.9	20,598	76.7+31.4	1,812
		> 1000 m	54.9+43.4	7,965	59.3+21.2	348

Table S2. Forest cover and aboveground carbon values (density and stock) for reserves of Hawaiian Island. These are only reserves that contain forested areas.

Name	Reserve type	Managing Agency	Island	Reserve Area (ha)	Forest Area (ha)	Aboveground Carbon Density (Mg C ha^{-1})	Aboveground Carbon Stock (Mg C)
Ahihi-Kinau Natural Area Reserve	State	DOFAW	Maui	836.7	31.1	5.8±5.7	179.7
Ahupuaa O Kahana State Park	State	DOSP	Oahu	2,110.0	1757.3	106.3±19.3	186830.1
Aiea Bay State Recreation Area	State	DOSP	Oahu	2.3	0.6	9.2±3.3	5.8
Akaka Falls State Park	State	DOSP	Hawaii	26.3	11.3	105.6±25.6	1198
Diamond Head State Monument	State	DOSP	Oahu	202.5	9.2	4.4±2.6	40.1
Ewa Forest Reserve (Poamoho Sec.)	State	DOFAW	Oahu	1,870.6	1480.1	90.7±30.3	134296.6
Ewa Forest Reserve (Waimano Sec.)	State	DOFAW	Oahu	1,031.3	875.3	92.7±26.0	81153.6
Haena State Park	State	DOSP	Kauai	95.5	21.7	119.9±25.6	2599.7
Hakalau Forest National Wildlife Refuge	Federal	USFWS	Hawaii	13,144.1	11739	94.5±45.2	1109518.9
Hakalau Forest National Wildlife Refuge (S.Kona Sec)	Federal	USFWS	Hawaii	2,165.4	1971.5	74.4±37.3	146670.7
Haleakala National Park	Federal	USNPS	Maui	13,597.3	4245.6	93.8±41.1	398220.5
Halekii-Pihana Heiau State Historic Site	State	DOSP	Maui	3.6	2.6	5.1±1.1	13.3
Halelea Forest Reserve	State	DOFAW	Kauai	6,068.8	3429.6	78.8±26.0	270090.4
Hamakua Forest Reserve (Kalopa Sec.)/ Kalopa State Recreation Area	State	DOFAW/DOSP	Hawaii	17.7	12.3	61.3±24.2	755.7
Hamakua Forest Reserve (Ahualoa Sec.)	State	DOFAW	Hawaii	128.1	42.6	68.9±14.8	2931.5
Hamakua Forest Reserve (Hanapai Sec.)	State	DOFAW	Hawaii	68.3	13.3	104.3±21.2	1389.4
Hamakua Forest Reserve (Hoea Kaao Sec.)	State	DOFAW	Hawaii	224.9	24.9	94.2±15.1	2349.2
Hamakua Forest Reserve (Honokaia Sec.)	State	DOFAW	Hawaii	197.3	7.5	76.3±31.9	569.8
Hamakua Forest Reserve (Kainehe Sec.)	State	DOFAW	Hawaii	15.5	1	99.8±12.5	98.8
Hamakua Forest Reserve (Kalopa Sec.)	State	DOFAW	Hawaii	194.9	14.8	97.5±18.0	1438.6

Name	Reserve type	Managing Agency	Island	Reserve Area (ha)	Forest Area (ha)	Aboveground Carbon Density (Mg C ha^{-1})	Aboveground Carbon Stock (Mg C)
Hamakua Forest Reserve (Kalopa Sec.)/ Kalopa Game Management Area	State	DOFAW	Hawaii	31.0	24.4	72.2±18.1	1759.9
Hamakua Forest Reserve (Keaa Sec.)	State	DOFAW	Hawaii	138.6	72.6	100.9±23.4	7331
Hamakua Forest Reserve (Paauilo Sec.)	State	DOFAW	Hawaii	422.1	262.5	98.4±18.5	25833.5
Hamakua Marsh Wildlife Sanctuary	State	DOFAW	Hawaii	9.1	2.3	28.1±14.5	65.8
Hana Forest Reserve	State	DOFAW	Maui	5,311.4	4204.8	52.2±19.8	219556.8
Hanalei National Wildlife Refuge	Federal	USFWS	Kauai	385.2	189.1	113.5±24.4	21457.7
Hanapepe Salt Ponds Historic Preserve	State	HPD	Kauai	5.0	0.3	11.7±5.3	3.2
Hanauma Bay State Underwater Park	State	DOSP	Oahu	40.9	0.4	13.3±1.8	4.8
Hanawi Natural Area Reserve	State	DOFAW	Maui	3,127.1	3037.9	70.1±32.6	213004.5
Hapuna Beach State Recreation Area	State	DOSP	Hawaii	24.8	6.6	11.2±8.7	73.6
Hapuna Beach State Recreation Area (Waialea Sec.)	State	DOSP	Hawaii	0.6	0.4	47.9±22.0	17.2
Hauola Forest Reserve	State	DOFAW	Hawaii	3.9	1.7	67.6±27.1	115.7
Hauula Forest Reserve	State	DOFAW	Oahu	572.9	313.9	107.9±24.3	33866.8
Hawaii Volcanoes National Park	Federal	USNPS	Hawaii	149,376.1	18563.8	48.7±34.5	903830.1
Heeia State Park	State	DOSP	Oahu	5.5	0.8	67.1±26.1	54.3
Hilo Forest Reserve (Humuula Sec.)	State	DOFAW	Hawaii	1,354.1	1305.9	133.0±32.6	173655.4
Hilo Forest Reserve (Kaiwiki Sec.)	State	DOFAW	Hawaii	2,087.2	1741.4	76.0±29.8	132283.5
Hilo Forest Reserve (Kamaee Sec.)	State	DOFAW	Hawaii	625.3	582.8	87.9±17.3	51236.9
Hilo Forest Reserve (Kauku Sec.)	State	DOFAW	Hawaii	27.4	0.4	107.4±10.5	38.7
Hilo Forest Reserve (Kukuau Sec.)	State	DOFAW	Hawaii	710.7	522.3	31.1±28.5	16249.7
Hilo Forest Reserve (Laupahoehoe Sec.)	State	DOFAW	Hawaii	1,961.1	1770.1	120.5±44.6	213223.2
Hilo Forest Reserve (Opea Sec.)	State	DOFAW	Hawaii	105.8	102.7	93.0±16.3	9547.3
Hilo Forest Reserve (Piha Sec.)	State	DOFAW	Hawaii	1,851.2	1829.1	105.4±38.7	192790.6

Name	Reserve type	Managing Agency	Island	Reserve Area (ha)	Forest Area (ha)	Aboveground Carbon Density (Mg C ha^{-1})	Aboveground Carbon Stock (Mg C)
Hilo Forest Reserve (Watershed Reserve Sec.)	State	DOFAW	Hawaii	16,984.5	15148.4	71.6±43.3	1084779.8
Hono O Na Pali Natural Area Reserve	State	DOFAW	Kauai	1,443.8	699.8	74.5±21.7	52138.9
Honolua-Mokuleia Marine Life Conservation District	State	DOAR	Maui	17.7	0.4	25.5±5.2	9.2
Honolulu Watershed Forest Reserve	State	DOFAW	Oahu	4,535.9	3347.4	96.0±26.0	321434.0
Honouliuli Forest Reserve	State	DOFAW	Oahu	1,433.5	569.5	75.1±29.5	42762.2
Honuaula Forest Reserve	State	DOFAW	Hawaii	3,442.2	2655.4	67.0±35.6	177840.3
Huleia National Wildlife Refuge	Federal	USFWS	Kauai	95.9	33.6	78.1±19.5	2621.4
Hulihee Palace	State	DOSP	Hawaii	0.5	0.3	25.2±14.9	6.8
Iao Valley State Monument	State	DOSP	Maui	2.8	1.7	78.7±16.1	134.5
Iolani Palace State Monument	State	DOSP	Oahu	4.3	0.9	19.2±2.5	17.3
James Campbell Nat Wildlife Refuge (Punamano)	Federal	USFWS	Oahu	15.0	2.9	21.7±10.4	62.4
James Campbell National Wildlife Refuge (Kii)	Federal	USFWS	Oahu	41.9	1.4	20.3±3.7	27.4
Kaala Natural Area Reserve	State	DOFAW	Oahu	439.1	287.8	65.1±21.2	18737.4
Kaena Point Natural Area Reserve	State	DOFAW	Oahu	30.2	2.4	9.8±5.6	23.8
Kaena Point State Park	State	DOSP	Oahu	630.8	102.4	8.6±9.8	883.7
Kahakuloa Game Management Area	State	DOFAW	Maui	498.4	314.4	48.4±28.0	15200.1
Kahaualea Natural Area Reserve	State	DOFAW	Hawaii	9,201.9	6447.5	90.0±29.1	580365
Kahikinui Forest Reserve	State	DOFAW	Maui	1,178.7	102.1	13.4±8.8	1368.9
Kahuku Motocross	Federal/ State	US Army/ DOFAW	Hawaii	346.8	222.2	74.8±32.7	16610.6
Kaipapau Forest Reserve	State	DOFAW	Oahu	392.5	322.5	92.4±23.3	29806.7
Kaiwi Scenic Shoreline	State	DOSP	Oahu	145.4	28.5	6.9±3.1	195.4
Kakahaia National Wildlife Refuge	Federal	USFWS	Molokai	20.3	4.5	33.8±16.8	152.2

Name	Reserve type	Managing Agency	Island	Reserve Area (ha)	Forest Area (ha)	Aboveground Carbon Density (Mg C ha^{-1})	Aboveground Carbon Stock (Mg C)
Kalaupapa National Historical Park	Federal	USNPS	Molokai	2,427.0	659.9	66.9±33.4	44175.4
Kalepa Mountain Forest Reserve	State	DOFAW	Kauai	272.3	109.4	75.7±28.6	8283.1
Kaloko-Honokohau National Historical Park	Federal	USNPS	Hawaii	480.9	34	17.1±11.4	582.6
Kaluanui Natural Area Reserve	State	DOFAW	Oahu	153.5	65.1	81.8±18.0	5320.7
Kamakou Preserve	Private	TNC	Molokai	1,065.5	648.7	67.2±18.7	43611.1
Kamehame Preserve	Private	TNC	Hawaii	12.6	2.2	17.5±3.0	37.9
Kanaele Preserve	Private	TNC	Kauai	26.6	2.2	51.4±21.2	111.1
Kanaha Pond Wildlife Sanctuary	State	DOFAW	Maui	58.1	1.7	29.1±16.5	49.8
Kanaio Natural Area Reserve	State	DOFAW	Maui	619.2	10	9.9±6.4	99.3
Kaneohe Forest Reserve	State	DOFAW	Oahu	26.2	11.3	112.9±25.3	1280.6
Kanepuu Preserve	Private	TNC	Maui	229.4	29.8	9.6±8.8	286.8
Kaohe Game Management Area	State	DOFAW	Hawaii	2,770.8	1319.6	4.8±3.1	6384.5
Kaohe Mitigation	State	DOFAW	Hawaii	609.7	11.3	2.6±0.9	29
Kapapala Cooperative Game Management Area	State	DOFAW	Hawaii	11,505.2	4621.1	64.0±37.9	295884.9
Kapapala Forest Reserve	State	DOFAW	Hawaii	15,084.8	2656.3	39.3±45.1	104342.7
Kapunakea Preserve	Private	TNC	Maui	542.1	397.3	70.0±21.5	27799.1
Kau Forest Reserve	State	DOFAW	Hawaii	24,945.5	23696.4	93.6±33.3	2216687.4
Kau Forest Reserve (Kamilo Section)	State	DOFAW	Hawaii	536.0	38.7	21.7±15.4	840.3
Kau Forest Reserve (Kapapala Sec.)	State	DOFAW	Hawaii	503.0	497.1	118.0±13.0	58642.1
Kau Preserve	Private	TNC	Hawaii	1,439.3	1241.5	78.5±22.8	97416
Kaumahina State Wayside	State	DOSP	Maui	2.9	2.9	140.2±19.4	403.9
Keaiwa Heiau State Recreation Area	State	DOSP	Oahu	156.1	87.7	112.0±23.9	9813.5
Kealakekua Bay State Historical Park	State	DOSP	Hawaii	89.6	4.7	32.6±11.1	152.8
Kealia Forest Reserve	State	DOFAW	Kauai	2,732.1	1784.4	88.7±25.1	158350.5
Kealia Pond National Wildlife Refuge	Federal	USFWS	Maui	281.1	58.3	37.6±16.8	2195

Name	Reserve type	Managing Agency	Island	Reserve Area (ha)	Forest Area (ha)	Aboveground Carbon Density (Mg C ha^{-1})	Aboveground Carbon Stock (Mg C)
Keauohana Forest Reserve	State	DOFAW	Hawaii	110.9	92.7	123.5±41.9	11444.6
Kekaha Game Management Area	State	DOFAW	Kauai	5,251.1	1061.9	43.1±35.1	45799.8
Kekaha Kai State Park	State	DOSP	Hawaii	676.2	98	7.0±7.5	685.7
Keolonahihi State Historical Park	State	DOSP	Hawaii	5.8	3.2	37.1±13.4	120.1
Keolonahihi State Historical Park/ Keakealaniwahine	State	DOSP	Hawaii	6.4	3	15.0±6.8	44.5
Kilauea Point National Wildlife Refuge	Federal	USFWS	Kauai	76.3	13.2	46.0±29.4	608.8
Kipahoehoe Natural Area Reserve	State	DOFAW	Hawaii	2,311.0	1042.4	42.0±22.3	43777.9
Kipahulu Forest Reserve	State	DOFAW	Maui	965.3	593.9	138.2±53.4	82068.2
Kipuka Ainahou Nene Sanctuary	State	DOFAW	Hawaii	14,260.1	1066.7	27.4±41.5	29176.4
Kohala Forest Reserve	State	DOFAW	Hawaii	3,201.8	2478.8	54.1±22.6	133988.1
Kohala Forest Reserve (Pololu Sec.)	State	DOFAW	Hawaii	678.4	217	93.7±26.0	20329.1
Kohala Forest Reserve (Waimanu Sec.)	State	DOFAW	Hawaii	1,865.0	1089.3	81.9±40.3	89195.2
Kohala Hist. Sites St. Monument (Kukuipahu Heiau)	State	DOSP	Hawaii	1.3	1	6.8±8.0	6.7
Kohala Watershed Forest Reserve	State	DOFAW	Hawaii	1,931.0	1532.1	61.2±28.6	93773.6
Kokee State Park	State	DOSP	Hawaii	1,775.8	1452.4	110.3±24.5	160261.4
Kona Hema Preserve	Private	TNC	Hawaii	3,281.1	2836.2	47.8±25.2	135543
Koolau Forest Reserve	State	DOFAW	Maui	12,589.2	11142.4	77.2±37.8	859781.9
Kuaokala Forest Reserve	State	DOFAW	Oahu	150.8	87.4	73.2±33.3	6398.9
Kuaokala Game Management Area	State	DOFAW	Oahu	771.2	377.6	38.5±31.0	14552.8
Kuia Natural Area Reserve	State	DOFAW	Kauai	639.5	443.3	107.9±21.2	47841.4
Kula Forest Reserve	State	DOFAW	Maui	1,983.0	923.6	81.2±37.4	74945.8
Kuliouou Forest Reserve	State	DOFAW	Oahu	86.7	34.4	67.9±19.0	2335.5
Lanai Cooperative Game Management Area	State	DOFAW	Lanai	13,792.7	5782.9	4.2±4.7	24199.8

Name	Reserve type	Managing Agency	Island	Reserve Area (ha)	Forest Area (ha)	Aboveground Carbon Density (Mg C ha^{-1})	Aboveground Carbon Stock (Mg C)
Lapakahi State Historical Park	State	DOSP	Hawaii	110.2	3.2	9.7±6.4	30.5
Laupahoehoe Natural Area Reserve	State	DOFAW	Hawaii	3,066.8	3037.6	118.6±41.8	360279.3
Lava Tree State Monument	State	DOSP	Hawaii	7.1	6.6	112.5±47.9	739
Lihue-Koloa Forest Reserve	State	DOFAW	Kauai	5,045.0	3309.8	88.2±27.9	291762
Lualualei Forest Reserve	State	DOFAW	Oahu	317.9	150.9	42.3±38.4	6389.5
Mackenzie State Recreation Area	State	DOSP	Hawaii	5.2	3.9	95.5±29.1	369.6
Makawao Forest Reserve	State	DOFAW	Maui	839.9	780.2	136.5±39.3	106525.9
Makena State Park	State	DOSP	Maui	68.4	23.1	23.0±12.9	532.1
Makiki Valley State Recreation Area	State	DOSP	Oahu	7.7	5	59.1±19.1	292.5
Makua Keaau Forest Reserve	State	DOFAW	Oahu	578.0	133.1	33.1±26.5	4404.2
Malaekahana State Recreation Area (Kahuku Sec.)	State	DOSP	Oahu	14.9	3.6	22.6±9.8	81.3
Malaekahana State Recreation Area (Laie Sec.)	State	DOSP	Oahu	29.8	14.4	40.2±17.2	578.7
Malama Ki Forest Reserve	State	DOFAW	Hawaii	612.9	445.9	65.1±37.3	29043.4
Mana Plains Forest Reserve	State	DOFAW	Kauai	55.7	7	18.1±6.2	127
Manowaialee Forest Reserve	State	DOFAW	Hawaii	650.6	571.2	130.9±18.0	74776.4
Manuka Natural Area Reserve	State	DOFAW	Hawaii	10,404.4	4004.6	39.3±19.2	157542.3
Manuka State Wayside	State	DOSP	Hawaii	5.4	2	51.5±25.9	102
Mauna Kea Forest Reserve	State	DOFAW	Hawaii	24,373.0	3846.2	4.1±2.1	15610.4
Mauna Kea Ice Age Natural Area Reserve	State	DOFAW	Hawaii	1,574.5	1.1	4.8±0.4	5.2
Mauna Kea State Recreation Area/ Mauna Kea Forest Reserve	State	DOFAW/DOSP	Hawaii	40.5	20.5	1.5±0.6	31.1
Mauna Loa Forest Reserve	State	DOFAW	Hawaii	22,183.9	9.3	23.0±14.7	213.4
Mokuleia Forest Reserve	State	DOFAW	Oahu	1,364.4	767	68.9±23.4	52830.7
Mokupuku Islet Sea Bird Sanctuary	State	DOFAW	Hawaii	1,385,375.1	712.9	71.9±48.8	51234.3

Name	Reserve type	Managing Agency	Island	Reserve Area (ha)	Forest Area (ha)	Aboveground Carbon Density (Mg C ha^{-1})	Aboveground Carbon Stock (Mg C)
Moloaa Forest Reserve	State	DOFAW	Kauai	1,515.6	975.2	102.6±28.2	100101.8
Molokai Forest Reserve	State	DOFAW	Molokai	4,115.7	2928.6	78.4±23.5	229650.9
Molokai Forest Reserve / Kalaupapa National Historical Park	State	DOFAW	Molokai	572.4	293.5	86.1±36.9	25278.8
Moomomi Preserve	Private	TNC	Molokai	374.7	209.7	6.2±4.1	1290.9
Nakula Natural Area Reserve	State	DOFAW	Maui	613.5	122.4	20.5±16.0	2511
Na Pali Coast State Wilderness Park	State	DOSP	Kauai	2,435.0	698.2	90.1±32.4	62872.8
Na Pali-Kona Forest Res/Alakai Wilderness Preserve	State	DOFAW	Kauai	4,205.0	3177.1	77.0±23.8	244615.1
Na Pali-Kona Forest Reserve	State	DOFAW	Kauai	5,116.5	2726.2	88.7±32.7	241858.6
Nanakuli Forest Reserve	State	DOFAW	Oahu	317.6	92.7	49.1±31.4	4551.6
Nanawale Forest Reserve	State	DOFAW	Hawaii	750.1	526.6	76.0±54.2	40014.2
Nanawale Forest Reserve (Halepuaa Sec.)	State	DOFAW	Hawaii	283.7	218.6	110.2±34.0	24094.9
Nounou Forest Reserve	State	DOFAW	Kauai	293.2	182.1	101.3±24.8	18442.4
Nuuanu Pali State Wayside	State	DOSP	Oahu	1.4	0.5	94.8±34.8	42.6
Oahu Forest National Wildlife Refuge	Federal	USFWS	Oahu	1,860.8	1581.7	81.9±25.2	129571.3
Olaa Forest Reserve	State	DOFAW	Hawaii	2,067.0	934.9	94.4±17.5	88295.4
Olaa Forest Reserve (Mt. View Sec.)	State	DOFAW	Hawaii	1,752.9	1684.3	64.8±24.0	109141.4
Olokui Natural Area Reserve	State	DOFAW	Molokai	680.9	544.1	81.8±23.9	44509
Ookala Cooperative Game Management Area	State	DOFAW	Hawaii	959.3	83.9	95.2±34.6	7980.8
Pahole Natural Area Reserve	State	DOFAW	Oahu	266.0	215.5	73.9±27.1	15925
Paiko Lagoon Wildlife Sanctuary	State	DOFAW	Oahu	15.9	0.5	21.0±7.8	11.3
Palaau State Park	State	DOSP	Molokai	94.1	87.6	96.9±19.7	8486.8
Pauwalu Point Wildlife Sanctuary	State	DOFAW	Maui	4.5	1.1	121.1±38.6	130.8
Pearl Harbor Nat'L Wildlife Refuge (Kalaeloa)	Federal	USFWS	Oahu	15.7	7.8	14.8±7.2	115.9

Name	Reserve type	Managing Agency	Island	Reserve Area (ha)	Forest Area (ha)	Aboveground Carbon Density (Mg C ha^{-1})	Aboveground Carbon Stock (Mg C)
Pearl Harbor National Wildlife Refuge (Mid Loch)	Federal	USFWS	Oahu	8.4	0.3	12.7±1.4	3.4
Pearl Harbor National Wildlife Refuge (W Loch)	Federal	USFWS	Oahu	12.2	0.2	28.0±0.0	5
Pelekunu Preserve	Private	TNC	Molokai	2,184.6	1351.7	74.4±21.1	100578.1
Pmrf Barking Sands	Federal	US Military	Kauai	823.7	304.8	16.1±7.0	4919.5
Pohakuloa Training Area Reservation	Federal	US Army	Hawaii	41,595.7	1366.7	1.0±1.5	1331.9
Pohakuloa Training Area Reservation (Keamuku Sec)	Federal	US Army	Hawaii	9,298.8	184.9	1.1±1.2	196.8
Pohakuloa Training Area Reservation/ Mauna Kea Forest Reserve	Federal/ State	US Army/ DOFAW	Hawaii	2,775.8	565	2.9±2.4	1638.1
Polihale State Park	State	DOSP	Kauai	59.7	2.2	35.0±15.8	75.5
Polipoli Spring State Recreation Area	State	DOSP	Maui	0.8	0.8	84.0±34.3	68.1
Pouhala Marsh Wildlife Sanctuary	State	DOFAW	Oahu	8.9	3.3	40.8±25.3	135.8
Puaa Kaa State Wayside	State	DOSP	Maui	0.7	0.4	79.0±27.3	28.4
Pupukea-Paumalu Forest Reserve	State	DOFAW	Oahu	316.8	239.9	109.5±22.6	26252.6
Puu Alii Natural Area Reserve	State	DOFAW	Molokai	534.6	426.6	84.9±21.3	36216.1
Puu Anahulu Game Management Area	State	DOFAW	Hawaii	25,508.8	1019.3	2.8±2.4	2825.8
Puu Honau O Honaunau National Historical Park	Federal	USNPS	Hawaii	73.6	3.1	14.6±8.6	44.6
Puu Ka Pele Forest Reserve	State	DOFAW	Kauai	9,454.3	2676.8	72.7±41.6	194631.8
Puu Ka Pele Forest Reserve/ Pacific Missile Range Facility Makaha Ridge	Federal/ State	US Military/ DOFAW	Kauai	83.1	29.2	23.2±16.4	675.6
Puu Kukui Watershed Preserve	Private	Maui Land and Pineapple Co.	Maui	2,948.9	2300.9	77.6±24.3	178489.3
Puu Makaala Natural Area Reserve	State	DOFAW	Hawaii	7,562.3	7112.6	92.3±36.4	656776.5

Name	Reserve type	Managing Agency	Island	Reserve Area (ha)	Forest Area (ha)	Aboveground Carbon Density (Mg C ha^{-1})	Aboveground Carbon Stock (Mg C)
Puu Mali Mitigation	State	DOFAW	Hawaii	2,050.7	411.8	2.7±2.6	1107.1
Puu O Mahuka Heiau State Historic Site	State	DOSP	Oahu	1.5	0.8	24.9±18.7	20.2
Puu O Umi Natural Area Reserve	State	DOFAW	Hawaii	4,262.0	3501.8	61.8±18.5	216382.8
Puu Ualakaa State Park	State	DOSP	Oahu	33.4	25.6	94.9±22.5	2425
Puu Waawaa Forest Bird Sanctuary	State	DOFAW	Hawaii	1,548.5	1098.6	50.2±20.2	55136.1
Puu Waawaa Forest Reserve	State	DOFAW	Hawaii	15,261.4	1089.7	10.1±12.8	10960.7
Puu Kohola Heiau National Historic Site	Federal	USNPS	Hawaii	33.9	3.5	20.2±13.1	71
Round Top Forest Reserve	State	DOFAW	Oahu	41.9	24.2	100.7±30.7	2437.5
Royal Mausoleum State Monument	State	DOSP	Oahu	2.8	2.1	76.8±25.1	159
Russian Fort Elizabeth State Historical Park	State	DOSP	Kauai	7.7	2.1	10.5±2.4	21.8
Sacred Falls State Park	State	DOSP	Oahu	402.7	240.7	104.8±19.8	25223.9
Sand Island State Recreation Area	State	DOSP	Oahu	58.6	18	17.0±6.0	306.3
South Kona Forest Reserve (Olelomoana Opihihali Sec.)	State	DOFAW	Hawaii	1,574.0	1158.9	59.8±33.8	69293.1
South Kona Forest Reserve (Kaohe Sec.)	State	DOFAW	Hawaii	619.0	400.3	59.5±37.3	23827.2
South Kona Forest Reserve (Kapua-Manuka Sec.)	State	DOFAW	Hawaii	1,090.7	916.7	65.1±25.6	59652.7
South Kona Forest Reserve (Kukuiopae Sec.)	State	DOFAW	Hawaii	1,147.4	1030.4	63.7±30.1	65678.1
Ulu Po Heiau State Historical Park	State	DOSP	Oahu	20.4	4.3	53.0±18.3	340
Upper Waiakea Bog Sanctuary	State	DOFAW	Hawaii	20.4	4.3	78.7±49.8	340
Upper Waiakea Forest Reserve	State	DOFAW	Hawaii	21,549.2	14929.7	61.8±35.9	923175.4
Waahila Ridge State Recreation Area	State	DOSP	Oahu	19.7	6.2	91.6±19.6	568.9
Wahiawa Freshwater State Recreation Area	State	DOSP	Oahu	27.0	14.5	96.3±22.4	1395.9
Waiaha Springs Forest Reserve	State	DOFAW	Hawaii	182.7	109.2	101.1±22.2	11031.2
Waiahole Forest Reserve (Iolekaa Sec)	State	DOFAW	Oahu	106.3	54.3	101.1±24.1	5488.6

Name	Reserve type	Managing Agency	Island	Reserve Area (ha)	Forest Area (ha)	Aboveground Carbon Density (Mg C ha^{-1})	Aboveground Carbon Stock (Mg C)
Waiahole Forest Reserve (Waiahole Sec)	State	DOFAW	Oahu	500.0	331.4	107.3±20.7	35544.6
Waiakea 1942 Lava Flow Natural Area Reserve	State	DOFAW	Hawaii	261.3	172.8	37.9±23.3	6541
Waiakea Forest Reserve	State	DOFAW	Hawaii	4,475.9	2611.3	71.6±28.0	186873.1
Waianae Kai Forest Reserve	State	DOFAW	Oahu	943.4	517.6	65.5±32.8	33901.9
Waianapanapa State Park	State	DOSP	Maui	41.4	12.1	101.9±25.7	1228.8
Waihee Coastal Dunes And Wetlands Refuge	Private	Maui Coastal Land Trust	Maui	116.6	65.2	35.0±19.5	2283
Waihou Spring Forest Reserve	State	DOFAW	Maui	76.8	74.7	126.7±36.4	9465.1
Waikamoi Preserve	Private	TNC	Maui	2,080.8	1318.3	101.4±29.7	133703
Waikamoi Preserve/ East Maui Irrigation	Private	TNC	Maui	1,433.6	1171.8	84.2±29.1	98610
Wailoa River State Recreation Area	State	DOSP	Hawaii	52.8	16.5	49.1±36.4	808.2
Wailua Game Management Area	State	DOFAW	Maui	686.0	587.2	96.8±19.6	56842.4
Wailua River State Park	State	DOSP	Kauai	428.2	2.4	79.7±53.1	22249.4
Wailua Valley State Wayside	State	DOSP	Maui	0.5	0.5	132.0±17.1	59.4
Wailuku River State Park	State	DOSP	Hawaii	6.3	2.4	79.7±53.1	193.6
Wailuku Silversword Sanctuary/ Mauna Kea Forest Reserve	State	DOFAW	Hawaii	49.5	4	5.0±1.0	19.8
Waimanalo Forest Reserve	State	DOFAW	Oahu	197.1	98	93.4±19.7	9149
Waimanalo Forest Reserve/ Mt Olomana State Monument	State	DOFAW	Oahu	84.9	53.4	113.6±13.8	6064.9
Waimea Canyon State Park	State	DOSP	Kauai	767.4	471.2	104.9±28.3	49424
Waimea State Recreation Pier	State	DOSP	Kauai	0.8	0.1	7.8±0.0	0.7
Wainiha Preserve	Private	TNC	Kauai	2,849.5	839.3	64.6±21.1	54232.9
Wao Kele O Puna Forest Reserve	State	DOFAW/OHA	Hawaii	10,433.7	7787.9	57.9±32.2	450849.9
West Maui Forest Reserve	State	DOFAW	Maui	4,604.0	1788.4	80.7±31.6	144284.8

Name	Reserve type	Managing Agency	Island	Reserve Area (ha)	Forest Area (ha)	Aboveground Carbon Density (Mg C ha^{-1})	Aboveground Carbon Stock (Mg C)
West Maui Natural Area Reserve (Honokowai Sec)	State	DOFAW	Maui	296.8	179.7	68.5±19.1	12313.1
West Maui Natural Area Reserve (Kahakuloa Sec)	State	DOFAW	Maui	1,415.9	532.3	83.2±25.5	44288.9
West Maui Natural Area Reserve (Lihau Sec)	State	DOFAW	Maui	373.4	40	63.2±28.5	2527.2
West Maui Natural Area Reserve (Panaewa Sec)	State	DOFAW	Maui	678.8	257.9	77.8±26.0	20059
Youth Challenge Academy	State	DOD State	Oahu	252.8	79.8	43.3±34.4	3455.8

USFWS - US Fish and Wildlife Service; USNPS - US National Park Service; DOFAW - Department of Forestry and Wildlife; DOSP - Division of State Parks; OHA - Office of Hawaiian Affairs; TNC – The Nature Conservancy; DOAR - Department of Aquatic Resources; DOD – Department of Defense.

Table S3: Species specific and general diameter-to-biomass models used to estimate aboveground biomass (AGB). D is diameter (cm) at 1.37 m from the ground or above buttress; H is height (m); ρ is wood density (g cm^{-3}); π is the ratio of a circle's circumference to its diameter. The general diameter-to-biomass model was applied when species-specific models were not available and when maximum D values for species-specific models were exceeded.

**Species**	**Diameter-to-AGB model (calculates AGB in kg)**	**Maximum D (cm)**	**Reference**
Acacia koa	exp(-2.3270+2.3500*ln(D))*1.0171	30.0	[14]
Metrosideros polymorpha	$0.2085*D^{2.318}$	33.0	[26]
Myrica faya	exp(-1.3412+2.1628*ln(D))	---	[14]
Psidium cattleianum	exp(-1.9096+2.5763*ln(D))*1.0084	18.2	[14]
Fraxinus uhdei (wood)[1]	exp(-2.7339+2.5974*ln(D))	91.6	[14]
Fraxinus uhdei (leaves)[1]	exp(-5.921+2.243*ln(D))	91.6	[14]
Cibotium spp.	$\pi*(D/2)^2*H*100*\rho/1000$	---	[14]
General	$0.0673*(\rho*(D^2)*H)^{0.976}$	212	[43]

[1]Results of models for wood and leaf tissues for individual trees were combined to estimate total AGB

Table S4: Species-specific and general diameter-to-height models used to estimate tree height when 1) species requiring the general diameter-to-biomass equation did not have tree height measured directly in the field and 2) when trees exceeded the maximum diameter for species-specific diameter-to-biomass models and tree height was not directly measured directly in the field. D is diameter (cm) at 1.37 m from the ground or above buttress; H is height (m); ρ is wood density (g cm^{-3}); E is a measure of environmental stress which incorporates temperature seasonality, climatic water deficit and precipitation seasonality (see [43]). All height-to-diameter models are from [14] except the general model, which is from [43].

Species	Region[1]	Diameter-to-height model (calculates H in m)	Max D (cm)	Max H (m)
Acacia koa	Dry	$\exp(0.0367+0.9886*\ln(D)-0.0871*\ln(D)^2)*1.0219$	130.2	20.4
Acacia koa	Wet	$\exp(0.1795+1.0160*\ln(D)-0.0800*\ln(D)^2)*1.0156$	140.4	28.0
Cibotium chamissoi	All	$\exp(0.6457+1.5932*\ln(D))*1.2763/100$	47.5	6.3
Cibotium glaucum	All	$\exp(-0.6277+1.6910*\ln(D))*1.1386/100$	58.0	6.0
Cibotium menziesii	All	$\exp(-0.6549+1.8683*\ln(D))*1.1705/100$	65.2	9.0
Cibotium spp.	All	$\exp(-0.4531+1.6955*\ln(D))*1.2071/100$	65.2	9.0
Metrosideros polymorpha	Dry	$14.1340*(1-\exp(-0.0573*D))$	98.3	21.5
Metrosideros polymorpha	Wet	$22.9975*(1-\exp(-0.0452*D))$	98.3	33.3
Psidium cattleianum	Wet	$12.4891*(1-\exp(-0.1569*D))$	26.7	15.3
General	All	$\exp(0.893-E+0.760*\ln(D)-0.034[\ln(D)]^2)$	130.2	21.5

[1]Dry indicates mean annual rainfall less than or equal to 1500 mm, Wet indicates mean annual rainfall greater than 1500 mm, All indicates the diameter-to-height model is applied across all rainfall regimes. Annual rainfall estimates for Hawaii are from Giambelluca et al. (2013).

Table S5: Estimates of wood density (WD, oven-dry mass divided by fresh volume in g cm^{-3}) for all trees and tree ferns (*Cibotium* spp.) present in FIA plots. N is the number of individuals of each species present across all FIA plots surveyed. Wood density values for each species are from [14]; WD values were averaged within a genus for trees with no specific epithet indicated in the FIA database, and a default WD value of 0.5 was used when no genus or species value from the literature was available.

Species	N	WD	Reference
Acacia confusa	85	0.73	[43]
Acacia koa	3172	0.55	[14]
Acacia spp.	10	0.77	[43]
Albizia chinensis	1	0.30	[43]
Aleurites moluccana	9	0.38	[43]
Antidesma platyphyllum	18	0.67	[14]
Antidesma ponapense	1	0.65	[43]
Antidesma pulvinatum	2	0.65	[43]
Archontophoenix alexandrae	4	0.83	[43]
Ardisia elliptica	74	0.60	[43]
Bambusa spp.	27	0.50	Default
Bobea sandwicensis	3	0.50	Default
Bocconia frutescens	1	0.50	Default
Broussaisia arguta	53	0.21	[14]
Casuarina cunninghamiana	1	0.70	[43]
Casuarina equisetifolia	79	0.81	[43]
Casuarina glauca	2	0.77	[43]
Cecropia obtusifolia	48	0.31	[43]
Cheirodendron trigynum	995	0.47	[14]
Chenopodium oahuense	3	0.50	Default
Chrysophyllum oliviforme	1	0.90	[43]
Cibotium chamissoi	68	0.21	[14]
Cibotium glaucum	1430	0.22	[14]
Cibotium menziesii	940	0.19	[14]
Cibotium spp.	62	0.21	[14]
Cinnamomum burmannii	28	0.49	[43]
Cinnamomum verum	12	0.50	[43]
Citharexylum caudatum	8	0.66	[43]
Clusia rosea	1	0.61	[43]
Cocos nucifera	11	0.50	Default
Coffea spp.	1	0.63	[43]
Colubrina oppositifolia	3	0.75	[43]

Coprosma longifolia	9	0.75	[43]
Coprosma montana	1	0.75	[43]
Coprosma ochracea	1	0.75	[43]
Coprosma rhynchocarpa	16	0.75	[43]
Coprosma spp.	77	0.75	[43]
Coprosma waimeae	1	0.75	[43]
Cordia subcordata	1	0.50	[43]
Cordyline fruticosa	10	0.50	Default
Cryptomeria spp.	319	0.36	[43]
Cyrtandra giffardii	1	0.50	Default
Diospyros sandwicensis	87	0.74	[14]
Erythrina sandwicensis	3	0.29	[43]
Eucalyptus deglupta	10	0.45	[43]
Eucalyptus grandis	248	0.66	[43]
Eucalyptus robusta	110	0.64	[43]
Eucalyptus saligna	1	0.74	[43]
Eucalyptus spp.	19	0.80	[43]
Falcataria moluccana	58	0.43	[14]
Ficus microcarpa	4	0.54	[43]
Ficus rubiginosa	25	0.43	[14]
Fraxinus uhdei	253	0.48	[14]
Fuchsia paniculata	1	0.70	[43]
Grevillea robusta	143	0.52	[43]
Hedyotis hillebrandii	6	0.38	[14]
Hedyotis terminalis	1	0.38	[14]
Heliocarpus popayanensis	17	0.24	[43]
Hibiscus tiliaceus	62	0.45	[43]
Ilex anomala	302	0.48	[14]
Jacaranda spp.	8	0.41	[43]
Leptospermum scoparium	2	1.03	[43]
Leucaena leucocephala	121	0.68	[43]
Macaranga mappa	2	0.27	[43]
Mangifera spp.	2	0.51	[43]
Melaleuca quinquenervia	25	0.63	[43]
Melaleuca spp.	12	0.70	[43]
Melicope clusiifolia	34	0.48	[43]
Melicope mucronulata	1	0.48	[43]
Melicope sandwicensis	15	0.48	[43]
Melochia spp.	6	0.32	[43]

Melochia umbellata	16	0.32	[43]
Metrosideros polymorpha	7753	0.69	[14]
Morella faya	21	0.66	[43]
Morinda citrifolia	23	0.63	[43]
Myoporum sandwicense	112	0.88	[43]
Myrsine lessertiana	243	0.53	[14]
Myrsine sandwicensis	16	0.53	[14]
Myrsine spp.	13	0.53	[14]
Nestegis sandwicensis	5	0.50	Default
Pandanus tectorius	67	0.33	[43]
Perrottetia sandwicensis	33	0.41	[14]
Persea americana	3	0.55	[43]
Phellodendron amurense	1	0.39	[43]
Pinus patula	31	0.42	[43]
Pinus pinaster	1	0.41	[43]
Pisonia brunoniana	4	0.34	[43]
Pithecellobium dulce	21	0.68	[43]
Pittosporum halophilum	2	0.62	[43]
Pittosporum hawaiiense	5	0.62	[43]
Pittosporum spp.	5	0.62	[43]
Pleomele spp.	3	0.50	Default
Pritchardia lanaiensis	1	0.50	Default
Pritchardia munroi	1	0.50	Default
Pritchardia spp.	1	0.50	Default
Prosopis pallida	32	0.88	[43]
Psidium cattleianum	1611	0.69	[14]
Psidium guajava	63	0.65	[43]
Psychotria hawaiiensis	97	0.54	[14]
Psychotria kaduana	4	0.56	[43]
Psychotria mariniana	7	0.56	[43]
Psychotria spp.	2	0.56	[43]
Psydrax odorata	10	0.87	[43]
Ptelea trifoliata	1	0.50	Default
Quercus robur	2	0.58	[43]
Rhodomyrtus tomentosa	1	0.78	[43]
Samanea saman	6	0.50	Default
Santalum paniculatum	6	0.77	[43]
Santalum spp.	8	0.77	[43]
Schefflera actinophylla	4	0.41	[43]

Schinus terebinthifolius	308	0.62	[43]
Sequoia sempervirens	19	0.38	[43]
Sophora chrysophylla	22	0.64	[43]
Spathodea campanulata	18	0.35	[43]
Syzygium cumini	87	0.67	[43]
Syzygium jambos	48	0.70	[43]
Syzygium malaccense	23	0.52	[43]
Syzygium sandwicense	1	0.65	[43]
Syzygium spp.	4	0.65	[43]
Toona ciliata	11	0.38	[43]
Tree broadleaf	56	0.50	Default
Tree evergreen	2	0.50	Default
Tree unknown	4	0.50	Default
Trema orientalis	5	0.35	[43]
Wikstroemia spp.	3	0.50	Default
